# Combined RNAseq and ChIPseq Analyses of the BvgA Virulence Regulator of Bordetella pertussis

**DOI:** 10.1128/mSystems.00208-20

**Published:** 2020-05-19

**Authors:** Loïc Coutte, Rudy Antoine, Stephanie Slupek, Luis Solans, Julien Derop, Amelie Bonnefond, David Hot, Camille Locht

**Affiliations:** a Université de Lille, CNRS, Inserm, CHU Lille, Institut Pasteur de Lille, U1019, UMR9017, CIIL, Center for Infection and Immunity of Lille, Lille, France; b Université de Lille, CNRS, CHU Lille, Institut Pasteur de Lille, UMR 8199, European Genomic Institute for Diabetes, Lille, France; University of Wisconsin—Madison

**Keywords:** *Bordetella pertussis*, RNAseq, ChIPseq, BvgA, response regulator

## Abstract

Bordetella pertussis regulates the production of its virulence factors by the two-component system BvgAS. In the virulence phase, BvgS phosphorylates BvgA, which then activates the transcription of virulence-activated genes (*vag*s). In the avirulence phase, such as during growth in the presence of MgSO_4_, BvgA is not phosphorylated and the *vag*s are not expressed. Instead, a set of virulence-repressed genes (*vrg*s) is expressed. Here, we performed transcriptome sequencing (RNAseq) analyses on B. pertussis cultivated with or without MgSO_4_ and on a BvgA-deficient Tohama I derivative. We observed that 146 genes were less expressed under modulating conditions or in the BvgA-deficient strain than under the nonmodulating condition, while 130 genes were more expressed. Some of the genes code for proteins with regulatory functions, suggesting a BvgA/S regulation cascade. To determine which genes are directly regulated by BvgA, we performed chromatin immunoprecipitation sequencing (ChIPseq) analyses. We identified 148 BvgA-binding sites, 91 within putative promoter regions, 52 within open reading frames, and 5 in noncoding regions. Among the former, 32 are in BvgA-regulated putative promoter regions. Some *vag*s, such as *dnt* and *fhaL*, contain no BvgA-binding site, suggesting indirect BvgA regulation. Unexpectedly, BvgA also bound to some *vrg* putative promoter regions. Together, these observations indicate an unrecognized complexity of BvgA/S biology.

**IMPORTANCE** Bordetella pertussis, the etiological agent of whooping cough, remains a major global health problem. Despite the global usage of whole-cell vaccines since the 1950s and of acellular vaccines in the 1990s, it still is one of the most prevalent vaccine-preventable diseases in industrialized countries. Virulence of B. pertussis is controlled by BvgA/S, a two-component system responsible for upregulation of virulence-activated genes (*vag*s) and downregulation of virulence-repressed genes (*vrg*s). By transcriptome sequencing (RNAseq) analyses, we identified more than 270 *vag*s or *vrg*s, and chromatin immunoprecipitation sequencing (ChIPseq) analyses revealed 148 BvgA-binding sites, 91 within putative promoter regions, 52 within open reading frames, and 5 in noncoding regions. Some *vag*s, such as *dnt* and *fhaL*, do not contain a BvgA-binding site, suggesting indirect regulation. In contrast, several *vrg*s and some genes not identified by RNAseq analyses under laboratory conditions contain strong BvgA-binding sites, indicating previously unappreciated complexities of BvgA/S biology.

## Abstract (precis)

Bordetella pertussis, the etiological agent of whooping cough, remains a major global health problem. Despite the global usage of whole-cell vaccines since the 1950s and of acellular vaccines in the 1990s, it still is one of the most prevalent vaccine-preventable diseases in industrialized countries. Virulence of B. pertussis is controlled by BvgA/S, a two-component system responsible for upregulation of virulence-activated genes (*vag*s) and downregulation of virulence-repressed genes (*vrg*s). By transcriptome sequencing (RNAseq) analyses, we identified more than 270 *vag*s or *vrg*s, and chromatin immunoprecipitation sequencing (ChIPseq) analyses revealed 148 BvgA-binding sites, 91 within putative promoter regions, 52 within open reading frames, and 5 in noncoding regions. Some *vag*s, such as *dnt* and *fhaL*, do not contain a BvgA-binding site, suggesting indirect regulation. In contrast, several *vrg*s and some genes not identified by RNAseq analyses under laboratory conditions contain strong BvgA-binding sites, indicating previously unappreciated complexities of BvgA/S biology.

## INTRODUCTION

Bordetella pertussis, the etiological agent of whooping cough, causes disease via the production of a number of virulence factors, including adhesins and toxins (1). Most of these factors are under the transcriptional control of the BvgA/S two-component system, of which BvgS is an inner membrane-spanning protein sensing environmental signals and BvgA is a cytoplasmic transcription factor (2). Via a BvgS-BvgA phosphorylation cascade, phosphorylated BvgA directly or indirectly activates the transcription of virulence-activated genes (*vag*s). They include genes coding for adhesins and toxins, as well as other virulence factors. In the absence of BvgA/S, *vag*s are not or are only minimally expressed, while the expression of another set of genes, collectively called virulence-repressed genes (*vrg*s), is strongly enhanced. The *vag*-*vrg* expression dichotomy can be observed in the laboratory by growing B. pertussis in the absence or presence of modulators, such as nicotinic acid or MgSO_4_, compounds that favor *vrg* expression at the expense of *vag* expression.

A number of studies using microarray (3–6) or, more recently, transcriptome sequencing (RNAseq) technologies (7, 8) have been carried out to define the Bvg regulon by identifying genes whose expression is down- (for the *vag*s) or upregulated (for the *vrg*s) by the addition of modulators or, in BvgA/S-deficient mutants compared to that in isogenic parent strains, when grown in the absence of modulators. However, these studies did not distinguish *vag*s that are directly regulated by BvgA through its binding to the promoter/operator regions from those that are indirectly regulated by BvgA via a regulatory cascade involving intermediate transcription factors. Examples for indirectly regulated genes include those that code for *Bordetella* type III secretion system (8).

In this study, we combined RNAseq and chromatin immunoprecipitation sequencing (ChIPseq) analyses to identify *vag*s together with BvgA-binding sites on the B. pertussis chromosome as a first step to decipher the global BvgA/S regulon cascade.

## RESULTS

### RNAseq analysis of the B. pertussis BvgA regulon.

RNAseq analyses were performed to decipher the global regulation of B. pertussis transcriptomes by BvgA according to its state of phosphorylation. RNA was isolated from the B. pertussis Tohama I derivative BPSM (9) grown in the absence or presence of 50 mM MgSO_4_ (BPSM Mg, referred to as the modulating condition) and from BPSMΔBvgA, a BPSM derivative carrying a genetic deletion of *bvgA* and in which *bvgS* is located directly downstream of the *bvgA/S* promoter. In the absence of MgSO_4_, BvgA reaches the maximal level of phosphorylation, although even under that condition, only approximately 50% of BvgA is phosphorylated (10). In the presence of MgSO_4_, BvgA is only present in its nonphosphorylated form (10). In BPSMΔBvgA, BvgA is not detectable, as expected (see Fig. S1 in the supplemental material).

10.1128/mSystems.00208-20.1 FIG S1 Detection of BvgA in cell lysates. Cellular lysates of BPSM, modulated BPSM (BPSM Mg), or BPSMΔBvgA were subjected to SDS-PAGE and immunoblotting with anti-BvgA antibodies. The arrow indicates the position of BvgA. The masses (in kDa) of the molecular markers are indicated at the left. Download FIG S1, PDF file, 0.4 MB. Copyright © 2020 Coutte et al. 2020 Coutte et al. This content is distributed under the terms of the Creative Commons Attribution 4.0 International license.

The global transcriptomic profiles of modulated BPSM and BPSMΔBvgA were very similar (Data Set S1, tab 1, Fig. S2). As expected, the expression of the *vag*s (green circle) was lower and that of the *vrg*s (red circle) was higher in modulated BPSM and in BPSMΔBvgA than in nonmodulated BPSM. The virtually identical overall transcriptomic profiles of modulated BPSM and BPSMΔBvgA suggest that essentially all *vag*s require phosphorylated BvgA to be expressed. The notable exception is *bipA*, the gene coding for intimin. The expression of this gene requires BvgA, as it was not expressed in BPSMΔBvgA, but its expression in BPSM was not affected by MgSO_4_ under the conditions tested here.

10.1128/mSystems.00208-20.2 FIG S2 Transcriptome comparisons of different strains under modulating and nonmodulating conditions. RNAseq RPKM gene expression ratios, expressed as log_2_ ratios, between the BvgA-depleted strain (BPSMΔBvgA) and the BPSM parental strain under modulating and nonmodulating conditions are depicted in the scatter plots. Each point represents one gene. The ratio between modulated and nonmodulated BPSM (*x* axis) is plotted against the ratio between BPSMΔBvgA and nonmodulated BPSM (*y* axis). Colored circles highlight genes of interest; green for the *vag*s and red for the *vrg*s; 50 mM MgSO_4_ was used as the modulating condition. Download FIG S2, PDF file, 0.6 MB. Copyright © 2020 Coutte et al. 2020 Coutte et al. This content is distributed under the terms of the Creative Commons Attribution 4.0 International license.

10.1128/mSystems.00208-20.9 DATA SET S1 Tab 1, Overall RNAseq results; tab 2, genes regulated as *vag*s or *vrg*s in the RNAseq results; tab 3, sRNA identified in the RNAseq results; tab 4, regulation of gene coding for putative B. pertussis regulators; tab 5, ChIPseq CLC Genomics peak caller results; tab 6, overall ChIPseq results; tab 7, comparison of MACS2 and CLC Genomics peak caller lists. Download Data Set S1, XLS file, 1.6 MB. Copyright © 2020 Coutte et al. 2020 Coutte et al. This content is distributed under the terms of the Creative Commons Attribution 4.0 International license.

**FIG 1:**
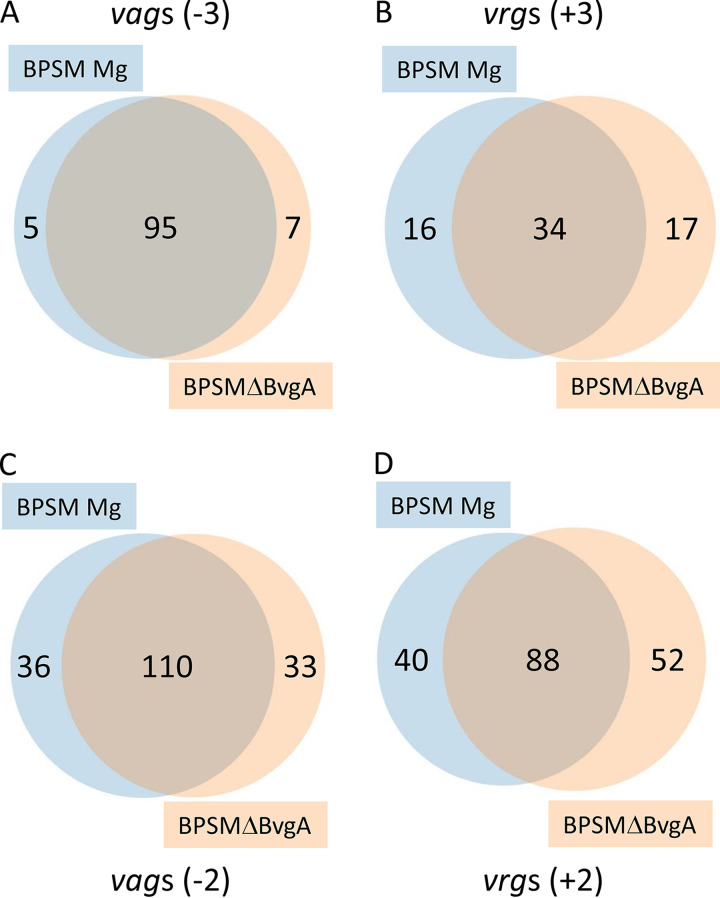
Venn diagrams showing the numbers of overlapping genes between BPSM Mg and BPSMΔBvgA. *vag*s(-3) (A) and *vag*s(-2) (C) are genes that present Log_2_FCs in transcription of less than −3 and −2, respectively, compared to that in BPSM. *vrg*s(+3) (B) and *vrg*s(+2) (D) are genes that present Log_2_FCs in transcription of >3 and >2, respectively, compared to that in BPSM.

To define the BvgA regulon using the comparative RNAseq data, we identified in BPSMΔBvgA and modulated BPSM genes that presented a Log_2_ fold change (Log_2_FC) in transcription less than −3 or greater than 3 compared to those in BPSM and designated them *vag*s(−3) and *vrg*s(+3), respectively (Data Set S1, tab 1). Rockhopper and SPARTA statistical analyses were performed to keep only statistically significant regulated genes with a *P* value of <0.05, as presented in Data Set S1, tab 2. In modulated BPSM, 100 *vags*(−3) and, in BPSMΔBvgA, 102 *vag*s(−3) were identified (Table 1; Data Set S1, tabs 1 and 2). By combining the two conditions, a total of 107 genes were identified as *vag*s(−3), 95 of which were in both BPSM Mg and BPSMΔBvgA (Fig. 1A). Furthermore, in modulated BPSM, 50 *vrg*s(+3) and, in BPSMΔBvgA, 51 *vrg*s(+3) were identified by comparison to nonmodulated BPSM. By combining the two conditions, 67 genes were identified as *vrg*s(+3), and 34 of them were identified in both BPSM Mg and BPSMΔBvgA, compared to those in nonmodulated BPSM (Fig. 1B).

**TABLE 1:** Summary of RNAseq analysis of the B. pertussis BPSM transcriptome

Category	Value		
	BPSM	BPSM Mg	BPSMΔBvgA
No. of genes			
*vag*s(−2)^*a*^		146	143
*vag*s(−3)^*a*^		100	102
*vag*s(−2) pseudogenes^*b*^	10	10	10
*vrg*s(+2)^*c*^		130	140
*vrg*s(+3)^*c*^		50	51
*vrg*s(+2) pseudogenes^*b*^	25	25	25
% of RPKM from:^*d*^			
*vag*s(−2)	18.45	2.03	4.52
*vag*s(−3)	8.31	0.27	0.4
*vrg*s(+2)	2.01	26.86	22.76
*vrg*s(+3)	0.6	22.77	17.27
*vag*s(−2) pseudogenes	0.55	0.06	0.03
*vrg*s(+2) pseudogenes	0.06	0.68	0.47
*vag*s(−3) pseudogenes	0.48	0.02	0.01
*vrg*s(+3) pseudogenes	0.02	0.51	0.35

a Genes presenting a *vag* profile with a Log_2_ fold change of less than −2 or −3, as indicated.

b Annotated as pseudogenes according to the Tohama I BX470248 genome annotation presenting a *vag* or *vrg* profile as indicated.

c Genes presenting a *vrg* profile with a Log_2_ fold change >2 or >3, as indicated.

d Percentage of the *vag*s(−2), *vag*s(−3), *vrg*s(+2), or *vrg*s(+3) and annotated pseudogene RPKM over the total RPKM from the complete Tohama I BX470248 genome ORF annotation.

When less-stringent threshold Log_2_FCs of −2 and +2 were used, the number of *vag*s and *vrg*s, named *vag*s(−2) and vrgs(+2), respectively, increased, as shown in the Table 1 and Fig. 1C and D. However, our statistical analysis showed more variability in the expression of these genes in the different samples. Therefore, at least for some of them, it may be questionable whether they are true *vag*s or *vrg*s regulated by the BvgA/S system.

Based on the calculated reads per kilobase per million (RPKM) corresponding to the *vag*s and *vrg*s under the different conditions, *vag*s(−3) and *vag*s(−2) represent 8.31% and 18.45%, respectively, of the total transcripts in BPSM (Table 1). These proportions decreased drastically in modulated BPSM and in BPSMΔBvgA. The *vrg*s(+3) represent 0.6% of the BPSM transcriptome, while they represent 22.77% and 17.27% of the modulated BPSM and the BPSMΔBvgA transcriptomes, respectively. As a reference, we used the housekeeping genes that present similar levels of transcription under all tested conditions (data not shown). The impact on the whole transcriptome of *vag*s and *vrg*s annotated as pseudogenes was also determined. Ten were annotated as pseudogenes among the *vag*s(−2), and only 3 were annotated as pseudogene among the *vag*s(−3), representing 0.56% and 0.49%, respectively, of the total transcripts in BPSM. Among the *vrg*s(+2), 25 were annotated as pseudogenes versus 11 among the *vrg*s(+3), and their expression represents between 0.01% and 0.68% of the total transcripts under the different conditions (Table 1). Thus, the total transcripts of the *vag* and *vrg* pseudogenes represent only a minor fraction of the entire transcriptome in B. pertussis.

### sRNA regulated by BvgA.

As noticed previously (11), the RNAseq data analyses also revealed transcripts corresponding to regions not annotated as coding for proteins (Data Set S1, tab 3). The analysis of all 6 data sets allowed us also to identify potential noncoding RNAs, most of which have already been seen in previous studies (7, 11). Among them, 90 were regulated under at least one tested condition (34 presenting a Log_2_FC of less than −2.27 presenting a Log_2_FC of less than −3.56 presenting a Log_2_FC of >2, and 30 presenting a Log_2_FC of >3). Most of them are 3′ or 5′ untranslated regions (UTRs) of *vags* or *vrgs*. However, 4 of them were not linked to the UTRs of *vag*s or *vrg*s and showed a *vrg*-type expression profile (Table 2). An additional noncoding RNA showed a *vag*-type expression profile (Table 2). Among these 5 potential noncoding RNAs, one had already been identified by Amman et al. (11) as candidate_transcript_301. It starts at the 3′ end of the *vrg bp2148* and extends into the open reading frame (ORF) of *bp2149* transcribed in the opposite orientation (see Fig. S3). Although transcript_301 is in the same orientation as *bp2148*, the two transcripts are probably not part of the same operon, as the expression level of transcript_301 is 2- to 3-fold higher than that of *bp2148*. A second one, corresponding to candidate_transcript_356 (11), is located between *bp2568* coding for a transposase of IS*481* and the 3′ end of *bp2569*. Its orientation is opposite that of *bp2568* and *bp2569*. A third transcript, highly transcribed in modulated BPSM and in BPSMΔBvgA, is located in the 3′ region of *bp2735*, a gene that is not part of the BvgA regulon. This transcript was recently named *rgtA* and shown to be involved in B. pertussis glutamate metabolism (12). We also detected the already documented transcript_242 (11) in the region upstream of *fim3*. This transcript was previously named *vrgX* (7, 13). Finally, we identified a transcript that was not previously described, starting at position 267174 and presenting a *vag-*like profile. It is close to the *bp0258* ATG start codon. *bp0258* is not a *vag* nor is its homologue *bb4499* in B. bronchiseptica. *bp0258* presented RPKMs in BPSM, BPSM Mg, and BPSMΔBvgA of 1388, 1232, and 1038, respectively, while for the new transcript, RPKM values were 133, 5, and 7 under the same respective conditions.

**TABLE 2:** Regulated sRNA identified in the B. pertussis BPSM RNAseq analysis

Name	Transcription site^*a*^		Strand^*b*^	RNA size (nt)	Synonym^*c*^	Rockhopper Log_2_FC^*d*^		Reference(s)^*c*^
	Start	Stop				BPSM Mg vs BPSM	BPSMΔBvgA vs BPSM	
Transcript_301	2270979	2271600	+	621		4.90	4.87	11
Transcript_356	2721117	2720970	+	147		5.12	5.72	11
*rgtA*	2903165	2903019	−	146	*rgtA*	3.55	3.52	12
Transcript_242	1647145	1647308	+	163	*vrgX*	3.12	3.98	7, 11, 13
Novel transcript	267174	267240	+	66		−4.73	−4.25	This study

a Transcription start and stop sites are the first and last nucleotides of the detected sRNA relative to the B. pertussis Tohama I BX470248 genome annotation.

b + and − represent forward and reverse directions, respectively, to the orientation of the B. pertussis Tohama I BX470248 genome annotation.

c Correspond to previously described sRNA.

d Log_2_FC, Log_2_ fold change of the RPKM of the detected sRNA under the tested condition versus BPSM.

10.1128/mSystems.00208-20.3 FIG S3 Schematic representation of the *bp2147-2149* locus. The blue arrows represent the gene sizes and orientation, and the orange arrow represents the size and orientation of candidate_transcript_301. Download FIG S3, PDF file, 0.5 MB. Copyright © 2020 Coutte et al. 2020 Coutte et al. This content is distributed under the terms of the Creative Commons Attribution 4.0 International license.

### BvgA, a regulator of regulators.

As shown previously (3, 7), BvgA is also involved in the regulation of several genes coding for proteins with putative regulatory functions. To evaluate how many regulatory genes are included in the BvgA regulon, we used the MiST2 database (http://mistdb.com) and identified 301 genes annotated in the B. pertussis genome coding for proteins with a putative regulatory function (Data Set S1, tab 4). Among them, 18 were identified as *vag*s(−2) or *vrg*s(+2) and 12 as *vag*s(−3) or *vrg*s(+3) (Table 3). Some of them had already been described and their function is known, such as *bvgS*, *bvgA*, and *brpL*. However, for most of them, the function remains unknown. These observations suggest that the BvgA regulon is composed of genes directly regulated by BvgA and of genes indirectly regulated by BvgA via a regulation cascade.

**TABLE 3:** Regulated genes coding proteins with putative regulatory functions.

Locus tag^*a*^	Gene product^*a*^	Rockhopper							SPARTA			
		Log_2_FC vs BPSM^*b*^		RPKM			*q* value vs BPSM		Log_2_FC vs BPSM^*b*^		*P* value vs BPSM	
		BPSM Mg	BPSMΔBvgA	BPSM	BPSM Mg	BPSMΔBvgA	BPSM Mg	BPSMΔBvgA	BPSM Mg	BPSMΔBvgA	BPSM Mg	BPSMΔBvgA
*bp0744*	Putative transcriptional regulator	−1.79	−2.18	424	123	94	0.00E+00	0.00E+00	−1.83	−1.97	1.26E−23	4.36E−04
*bp0764*	Probable LysR-family transcriptional regulator	−4.17	−4.17	18	0	0	4.82E−02	2.74E−01	−4.84	−3.76	3.13E−44	1.77E−07
*bp1496*	Probable two-component response regulator	−4.48	−4.26	134	6	7	1.00E+00	1.00E+00	−4.50	−4.06	5.02E−65	1.09E−09
*bp1798*	Putative DNA-binding protein	0.82	2.07	210	371	879	0.00E+00	4.41E−75	0.81	2.62	8.45E−06	3.82E−04
*bp1876*	Regulatory protein BvgR	−6.38	−6.39	919	11	11	1.00E+00	1.00E+00	−5.91	−5.75	1.24E−129	1.52E−17
*bp1877*	Virulence sensor protein	−2.02	−1.78	271	67	79	1.00E+00	1.00E+00	−1.85	−1.57	1.04E−21	5.16E−03
*bp1878*	Virulence factor transcription regulator	−2.29	−3.39	3331	679	318	1.00E+00	1.00E+00	−2.03	−2.75	3.61E−22	1.62E−04
*bp2142*	Putative GntR-family transcriptional regulator	−1.83	−2.68	32	9	5	2.81E−05	7.46E−141	−1.69	−2.27	1.83E−12	5.10E−04
*bp2227*	Putative anti-sigma factor	−3.89	−3.89	74	5	5	1.00E+00	1.00E+00	−3.68	−3.30	3.53E−39	3.38E−07
*bp2230*	Putative regulator	−4.46	−4.46	22	0	0	1.00E+00	1.00E+00	−4.80	−4.13	9.34E−62	5.56E−09
*bp2234*	Putative RNA polymerase sigma factor	−8.00	−8.59	770	3	2	1.00E+00	1.00E+00	−7.82	−7.46	2.51E−175	3.45E−24
*bp2436*	Putative membrane protein	0.50	2.63	358	506	2213	8.19E−07	1.00E+00	0.54	3.26	1.24E−02	3.85E−06
*bp2437*	RNA polymerase sigma factor	0.18	2.13	641	727	2805	2.13E−05	3.19E−11	0.11	2.12	5.64E−01	2.49E−03
*bp2571*	Putative transcriptional regulatory protein	1.40	2.01	429	1133	1722	2.63E−01	4.64E−02	1.41	2.37	1.44E−12	1.10E−04
*bp2635*	Leucine-responsive regulatory protein	1.75	2.44	70	235	380	1.00E+00	1.00E+00	1.68	2.78	7.76E−19	4.24E−06
*bp2872*	Putative AsnC-family transcriptional regulator	2.24	0.51	7	33	10	1.74E−148	7.90E−24	2.06	0.76	6.91E−09	1.95E−01
*bp2935*	Putative two-component system, histidine kinase	−2.81	−2.09	98	14	23	5.58E−01	1.44E−03	−2.97	−2.15	1.51E−39	2.60E−04
*bp3702*	GntR-family transcriptional regulator	2.02	2.36	33	134	170	4.38E−02	2.66E−01	1.96	2.61	5.87E−25	1.72E−06

a According to B. pertussis Tohama I BX470248 genome annotation.

b Log_2_FC, Log_2_ fold change of the RPKM of the select genes under the tested condition versus BPSM.

### Analysis of the *bvgA* promoter region.

**FIG 2:**
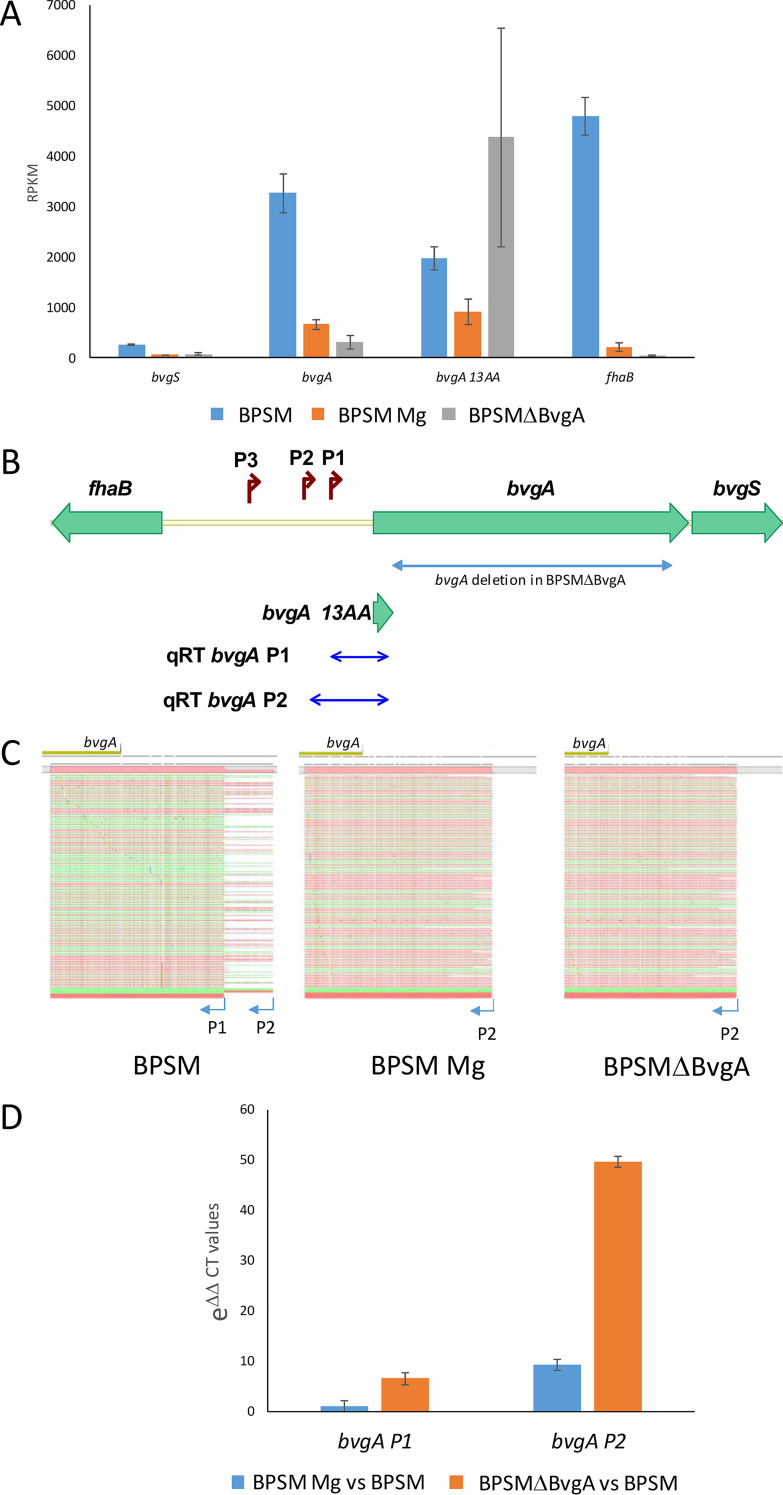
Transcription profiling, RACE, and qRT PCR analysis on the *bvgA* promoter region. (A) RPKM quantification in BPSM, modulated BPSM (BPSM Mg), and BPSMΔBvgAfor *bvgS*, *bvgA*, and *fhaB*. *bvgA13AA* corresponds to the region coding for the 13 first amino acids of BvgA. RPKM standard deviations (SD)s are shown. (B) Schematic representation of the B. pertussis *fhaB*-*bvgAS* promoter region. P1, P2, and P3 represent the described *bvgA* promoters. qRT *bvgA* P1 and P2 depict the regions used to quantify the promoter strength of P1 and P2, respectively, by qRT PCR. (C) Mapping of the RACE reads obtained with the BvgA RACE primer on BPSM, modulated BPSM (BPSM Mg), and BPSMΔBvgA RNA. Single reads mapping in the forward direction are in green. Single reads mapping in the reverse direction are red. Any mismatches between the reads and the reference sequence are shown as colored dots. (D) Quantitative RT-PCR analysis of P1 and P2 promoter regions of *bvgA*. mRNA transcripts corresponding to the *bvgA* promoters P1 and P2, as shown in B, were amplified by qRT-PCR using a *bvgA*-specific reverse primer and a primer corresponding to either P1 or P2 (see panel B). Expression of *bvgA* P1 and P2 transcripts is presented relative to the level of expression of the housekeeping gene *bp3416*.

A focus on the *bvg*-*fha* locus indicated that the expression of *bvgA* was reduced 4.89- and 10.48-fold in modulated BPSM and in BPSMΔBvgA, respectively, compared to that in nonmodulated BPSM. That of *bvgS* was reduced 4.02- and 3.41-fold, respectively, and that of *fhaB* was reduced 22.31- and 121.93-fold, respectively (Fig. 2A). We were intrigued by the observation that in the RNAseq analysis, the reads comprising the promoter region and first codons of *bvgA* were much more abundant in BPSMΔBvgA than in BPSM and BPSM Mg. BPSMΔBvgA contains an in-frame deletion fusing the first 13 codons to the last 7 codons of *bvgA*, hence coding for a nonfunctional BvgA truncate. We therefore measured specifically the RPKM corresponding to the first 13 *bvgA* codons under all tested conditions and found that the transcripts of this region were 2.21- and 4.78-fold more abundant in BPSMΔBvgA than in BPSM and BPSM Mg, respectively. Previous studies have shown that the region between the divergently transcribed *bvgAS* and *fhaB* genes contains at least three promoters that control *bvgA* expression (Fig. 2B) (14). The transcriptional start sites of promoters P1, P2, and P3 were reported to be located 93 bp, 143 bp, and 272 bp, respectively, upstream of the *bvgA* translational start site. Although we did not find any read corresponding to P3 in our RNAseq analysis, we detected many reads corresponding to P1 and P2. To determine which promoter(s) was used to drive *bvgA* expression under the various conditions, we performed 5′ rapid amplification of cDNA ends (RACE) experiments on the 5′ UTR of *bvgA* (Fig. 2C) and found that in nonmodulated BPSM, *bvgA* expression was mainly driven by P1, although some reads were also detected coming from P2. In contrast, in BPSM Mg and BPSMΔBvgA *bvgA* expression was exclusively driven by P2. To quantify the strength of P1 and P2 under each condition, reverse transcription-quantitative PCR (qRT-PCR) experiments were performed (Fig. 2D) and showed that the transcripts corresponding to P2 were 9.27-fold more abundant in BPSM Mg than in BPSM and 49.67-fold more abundant in BPSMΔBvgA than in BPSM. These results indicate differential usage of the *bvgA* promoters under the three conditions.

### ChIPseq analysis of BvgA binding in B. pertussis.

To identify *vag*s that may be directly regulated by BvgA, ChIPseq analyses were performed to localize the BvgA-binding sites on the genome of B. pertussis. Among the 2,055 signals initially identified by CLC Genomics peak caller using all 6 data sets (2 for BPSM, 2 for BPSM Mg, and 2 for BPSMΔBvgA) (Data Set S1, tab 5), we selected those with a peak shape score of >5 and an associated *P* value of ≤3.79 × 10^43^, which revealed 148 BvgA-binding sites in BPSM (Data Set S1, tab 6). None of them were detected in BPSM Mg or BPSMΔBvgA, indicating strong specificity of the assay and showing that the ability of BvgA to bind to its cognate DNA sites depends on its phosphorylation. Ninety-one BvgA-binding sites were found in putative promoter regions of annotated ORFs (first category) (see examples in Fig. S4A and B). Putative promoter regions are defined here as regions upstream of an ORF spanning between −5 and −806 nucleotides (nt) from the predicted translational initiation site and not overlapping with an adjacent ORF. Most of the BvgA-binding sites found in these regions are located between nucleotides −150 and −1 from the predicted ATG translational start site (data not shown). Fourteen of the BvgA-binding sites of the first category were found in the putative promoter regions located between two ORFs orientated in opposite directions (Table 4; Fig. S4B). Five were found in noncoding regions (second category) (Fig. S4C) and 52 within annotated ORFs (third category) (Fig. S4D and E). Among the latter, 14 are located close to the 5′ end of an adjacent ORF and could therefore be part of the promoter/operator region of the downstream gene (Fig. S4E). We also applied MACS2 genomics peak caller comparing the BPSM and the BPSMΔBvgA run data sets. Using a *P* value threshold of 1.00 e^−5^, 143 peaks were identified, and all of them were also found in the list established with CLC peak caller (Data Set S1, tab 7). The additional peaks identified by CLC correspond to peaks located within insertion sequences, which were deliberately excluded in the MACS peak caller analysis.

**TABLE 4:** Summary of ChIPseq analysis of the B. pertussis BvgA regulator

Category	No. of genes with BvgA-binding site in:					
	Putative promoter	Promoter between^*a*^	Non-coding^*b*^	Inside^*c*^	Promoter or inside^*d*^	Total
*vags*^*e*^	23	0	0	6	0	29
Additional *vags*^*f*^	8	0	0	1	1	10
*vrg* RisA independent^*g*^	3	0	0	1	1	5
*vrg* RisA dependent^*g*^	2	1	0	2	0	5
RisA regulated^*h*^	1	0	0	0	0	1
Bvgi^*i*^	1	0	0	0	0	1
Non-coding^*j*^	0	0	5	0	0	6
No *vag* no *vrg*^*k*^	39	12	0	28	12	91
Total	77	14	5	38	14	148

a Putative promoter region located between two ORFs orientated in opposite directions.

b Region not annotated in the B. pertussis Tohama I BX470248 genome.

c Within annotated ORF of the B. pertussis Tohama I BX470248 genome.

d Within annotated ORF and close to the 5′ end of an adjacent ORF.

e *vags* presenting a Log_2_FC of less than −2 in the RNAseq analysis.

f *vags* presenting a Log_2_FC between −1.5 and −2 in the RNAseq analysis.

g *vrg*s presenting a Log_2_FC of >2 in the RNAseq analysis and were described by Coutte et al. (3).

h RisA-regulated gene, *bp1022*, codes for the transcriptional activator FlbB, described by Coutte et al. (3).

i Bvg^i^ corresponds to *bipA*, not regulated by modulation under the conditions tested here but requires BvgA.

j Regions not annotated in the B. pertussis Tohama I BX470248 genome.

k Gene not regulated as *vag* or *vrg* in this RNAseq analysis.

10.1128/mSystems.00208-20.4 FIG S4 Representation of the different localizations of the putative BvgA-binding sites identified by ChIPseq. (A) Example of BvgA-binding site found in putative promoter region of annotated ORF. (B) Example of BvgA-binding site found in the putative promoter regions located between two ORFs orientated in opposite directions. (C) Example of BvgA-binding site found in noncoding region. (D) Example of BvgA-binding site found within annotated ORF. (E) Example of BvgA-binding site found within annotated ORF and located close to the 5′ end of an adjacent ORF. The yellow arrows represent the gene orientation. Single reads mapping in their forward direction are in green. Single reads mapping in their reverse direction are red. Any mismatches between the read and reference are shown as colored dots. More reads are mapped to the reference than can be shown in detail; so, these reads are displayed in an overflow graph below the reads. The overflow graph is shown in the same colors as the reads, and mismatches in reads are shown as colored narrow vertical lines within the overflow graph. Download FIG S4, PDF file, 0.7 MB. Copyright © 2020 Coutte et al. 2020 Coutte et al. This content is distributed under the terms of the Creative Commons Attribution 4.0 International license.

**FIG 3:**
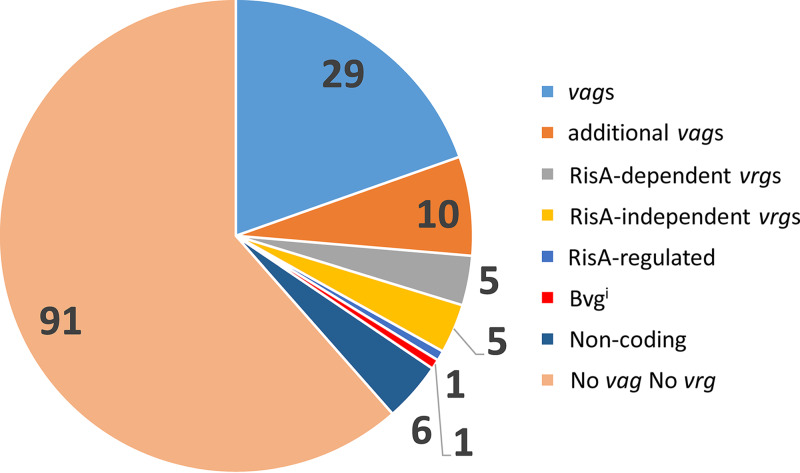
Graphical representation of the different BvgA-binding sites with respect to identified BvgA-regulated genes on the B. pertussis genome. Numbers correspond to the numbers of BvgA-binding sites in each category.

A comparison of the RNAseq data using Log_2_FC of less than −2 as the cutoff with the ChIPseq data indicated that among the 148 BvgA-binding sites, 29 are located closed to *vag*s (23 in putative promoter regions and 6 within ORFs) (Table 4 and Fig. 3). In addition, some genes with a BvgA-binding site also showed a *vag*-like profile, with a Log_2_FC between −1.5 and −2. Ten of them showed a BvgA-binding site (9 in putative promoter regions and 1 within ORF). Ten BvgA-binding sites were found located close to *vrgs*, 5 of which were previously identified as being RisA dependent and 5 as RisA independent (3). The RisA-regulated gene identified in Table 4 and Fig. 3 has a BvgA-binding site close to *bp1022*, coding for the transcriptional activator FlbB involved in the transcription of the flagellar operon that does not depend on BvgA but is regulated by BvgR and RisA (3). Furthermore, *bipA*, a gene that is not regulated by modulation under the conditions tested here but requires BvgA (Table 4 and Data Set S1, tab 1), also contains a BvgA-binding site, called Bvg^i^ in Fig. 3 and in Data Set S1, tab 6.

Six BvgA-binding sites were found located either between the 3′ ends of two convergent genes or in a region upstream of *fim3*, called *vrgX*, and identified as transcript 151 in the RNAseq data (7, 13). We also identified a BvgA-binding site close to the novel transcript upstream of *bp0258*, confirming the *vag-*like profile of this small RNA (sRNA). None of the other putative sRNA identified by RNAseq were associated with a BvgA-binding site. The remaining 91 BvgA-binding sites located close to ORFs were not found to be regulated as *vag*s or *vrg*s in our RNAseq data (Table 4).

A comparison of ChIPseq and RNAseq data is presented as a scatterplot in Fig. S5. Genes immediately downstream of a ChIPseq peak or with an internal ChIPseq peak present a *vag*, a *vrg*, or an unregulated profile of regulation in our study, indicating the diversity of BvgA regulation.

10.1128/mSystems.00208-20.5 FIG S5 Scatterplot representation of the RNAseq and ChIPseq data comparison. Regulation of the 91 genes presenting BvgA-binding sites found in putative promoter regions of annotated ORFs are represented in black and regulation of the 52 genes presenting BvgA-binding sites found in within ORFs are represented in red. RNAseq RPKM gene expression ratios, expressed as log_2_ ratios, between BPSMΔBvgA and BPSM parental strain (*y* axis) and modulated and nonmodulated BPSM (*x* axis). Each point represents one gene; 50 mM MgSO_4_ was used as the modulating condition. Download FIG S5, PDF file, 0.4 MB. Copyright © 2020 Coutte et al. 2020 Coutte et al. This content is distributed under the terms of the Creative Commons Attribution 4.0 International license.

The 107 genes identified as *vag*s by our RNAseq analyses are grouped into 42 different transcription units, 23 of which contain strong BvgA-binding sites (Table 4; Fig. S5). Three additional ones contained a ChIPseq peak in their promoter region with less than 1,000 reads used as threshold (*bp2486* with 850 reads; *bp2738*/*bapC* with 950 reads and *bp3619* with 979 reads). However, the reads corresponding to these regions were much more abundant in BPSM than under the other conditions, suggesting that they indeed contain true BvgA-binding sites. *fim2* and *fim3* did not present an automatically detectable ChIPseq peak, likely due to the informatic processing of the Illumina reads that removes reads with long stretches of cytosines. In fact, both genes present atypical ChIPseq peaks in their promoter region, but the numbers of reads are consistent with them having a BvgA-binding site (see Fig. S6). Finally, 16 transcription units of *vag*s did not present any ChIPseq peak, suggesting that they are not directly regulated by BvgA. They are *bp0399*, *bp0499*, *bp0500*, *bp0456* (*hemC*), *bp0535*, *bp2147*, *bp2226*, *bp2227*, *bp2232, bp2233*, *bp2256, bp2257, bp2749*, *bp2907* (*fhaL*), *bp3433*, and *bp3439* (*dnt*), identified as *vag*s(−3) in the RNAseq analysis.

10.1128/mSystems.00208-20.6 FIG S6 Representation of the ChIPseq reads mapping on the *fim2* and *fim3* loci. (A) ChIPseq-mapped reads on the *fim2* locus on BPSM ChIPseq run 1 (top) and BPSM ChIPseq run 2 (bottom). (B) ChIPseq-mapped reads on the *fim3* locus on BPSM ChIPseq run 1 (top) and BPSM ChIPseq run 2 (bottom). The yellow arrows represent the gene orientation. Single reads mapping in their forward direction are in green. Single reads mapping in their reverse direction are red. Any mismatches between the read and reference are shown as colored dots. More reads are mapped to the reference than can be shown in detail. Therefore, these reads are displayed in an overflow graph over the reads. The overflow graph is shown in the same colors as the reads, and mismatches in reads are shown as colored narrow vertical lines within the overflow graph. Download FIG S6, PDF file, 0.7 MB. Copyright © 2020 Coutte et al. 2020 Coutte et al. This content is distributed under the terms of the Creative Commons Attribution 4.0 International license.

Therefore, we examined the ChIPseq data to identify BvgA-binding sites close to genes coding for proteins with regulatory functions. Among the 301 genes annotated in the B. pertussis genome that code for proteins with a putative regulatory function, 15 presented a BvgA-binding site in their promoter region or belong to an operon containing a BvgA-binding site (see Fig. S7) and would therefore be candidates as intermediate regulators of certain *vag*s.

10.1128/mSystems.00208-20.7 FIG S7 Heat map of expression of regulatory genes presenting BvgA-binding sites. The genes, products, and synonyms correspond to the Tohama I Sanger Centre annotation. BPSM Mg versus BPSM corresponds to the Log_2_FC RPKM ratios between BPSM cultivated in the presence of 50 mM MgSO_4_ and in nonmodulated BPSM. BPSMΔBvgA versus BPSM corresponds to the Log_2_FC RPKM ratios between BPSMΔBvgA and nonmodulated BPSM. The indicated ratios are the means from all the experiments. Red, increased transcript abundance; green, decreased transcript abundance; black, no significant change in transcript abundance; the level of transcript abundance is defined by the colored Log_2_FC scale shown on the bottom. Download FIG S7, PDF file, 0.5 MB. Copyright © 2020 Coutte et al. 2020 Coutte et al. This content is distributed under the terms of the Creative Commons Attribution 4.0 International license.

### Search for BvgA binding consensus.

**FIG 4:**
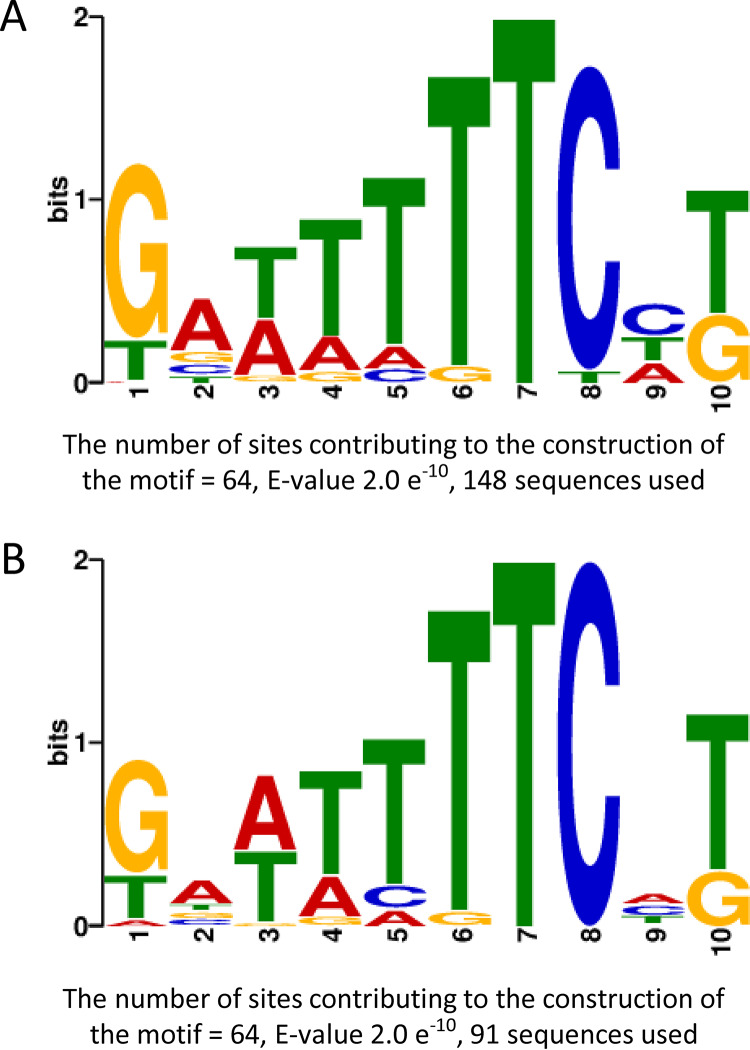
MEME analysis of motifs identified in the ChIPseq peaks. (A) Motif found using the 148 putative BvgA-binding sites. (B) Motif found using the 91 putative BvgA-binding sites located in promoter regions.

In an attempt to identify BvgA-binding consensus sequences based on the ChIPseq data, we used MEME ChIPseq software with the “zero or one occurrence per sequence” or “any number of repetitions” options to analyze the regions showing BvgA binding. For each binding site, we considered the center of the peak detected in ChIPseq BPSM run 1 and 100 nucleotides on each side to define a 201-bp region. We clustered them in 3 categories, binding at any position (Fig. 4A) (*n* = 148), binding in putative promoter regions (Fig. 4B) (*n* = 91), and binding within ORFs. In using the regions corresponding to the 148 BvgA-binding sites or the 91 sites located in putative promoter regions, a strong and consensus motif was found using the “zero or one occurrence per sequence” option (Fig. 4A and B). This 10-bp motif is similar to the BvgA-binding site consensus already described (15). Using the 52 regions comprising sites within ORFs, we were not able to identify a statistically relevant consensus.

## DISCUSSION

This study presents a combined RNAseq and ChIPseq analysis of the BvgA regulon of B. pertussis to identify the transcriptome of B. pertussis directly or indirectly regulated by BvgA.

A comparison of our RNAseq data with those of a previous study (7) showed that while most *vag*s and *vrg*s identified here were also identified by Moon et al. (7) (see Fig. S8), some differences remained. They could be due to (i) use of different Tohama I derivatives between the two studies, (ii) differences in fold change threshold levels used to identify *vag*s and *vrg*s, (iii) the use of different culture media in the two studies, (iv) annotation errors, and (v) use of a BPSM BvgA mutant versus a BP536 BvgA/S double mutant. As an example of an annotation error, *bp1055* was considered *vag* by Moon et al. (7) but not in this study because of reads overlapping from the 3′ end of the *vag bp1054* (*prn*) to *bp1055*, which is not a *vag* in our study (Fig. S8A).

10.1128/mSystems.00208-20.8 FIG S8 Scatterplot representations of RNAseq data comparisons. Comparison of the RNAseq RPKM gene expression ratios, expressed as log_2_ ratios, between the data set of Moon et al. (7) and the RNAseq data set of this study. (A) The ratio between modulated and nonmodulated BPSM (*x* axis; this study) is plotted against the ratio between modulated and nonmodulated BP536 (*y* axis; Moon et al. [7]). (B) The ratio between BPSMΔBvgA and the nonmodulated BPSM parental strain (*x* axis; this study) is plotted against the ratio between BP536ΔBvgAS and the nonmodulated BP536 parental strain (*y* axis; Moon et al. [7]). RPKM gene expression ratios are expressed as log_2_ ratios; 50 mM MgSO_4_ was used as the modulating condition for both studies. Download FIG S8, PDF file, 0.6 MB. Copyright © 2020 Coutte et al. 2020 Coutte et al. This content is distributed under the terms of the Creative Commons Attribution 4.0 International license.

Together with a few housekeeping genes, the *vag*s are among the most highly expressed genes under nonmodulating conditions, representing almost 20% of the transcriptome. Under modulating conditions or in BPSMΔBvgA, the *vrg*s are also highly expressed, representing almost one-quarter of the transcriptome. These observations indicate that expression control by the BvgA/S two-component system has a major impact on overall transcriptional activity in B. pertussis. In contrast to that of functional *vag*s and *vrg*s, the expression of *vag* and *vrg* pseudogenes plays a minor role in the overall transcriptomic activity of B. pertussis.

The RNAseq analysis allowed us also to identify 5 putative sRNAs regulated by BvgA, which could not be identified in previous DNA microarray studies (3, 4), as these studies only considered annotated ORFs of the B. pertussis chromosome. The most highly expressed and tightly regulated sRNA, *rgtA*, was recently shown to play a role in B. pertussis glutamate metabolism, linking the BvgA regulon to an important metabolic pathway, as glutamate is the main carbon source for B. pertussis in culture media (12). A BvgA-binding site was detected upstream of a novel transcript with a *vag*-type expression profile, confirming its regulation by BvgA. Additional experiments will be required to confirm the remaining sRNAs and to decipher their roles in B. pertussis biology or pathogenesis.

The regulation of *bvgA* expression itself is complex and is both BvgA dependent and independent. Promoter P1 is highly active in the virulent phase (nonmodulated BPSM), while P2 is mostly used in the avirulent phase (during modulation or in BPSMΔBvgA). This mechanism may be used by B. pertussis to ensure an almost constant level of BvgA regardless of the virulence phases, as seen by immunoblot analyses (Fig. S1), even in the absence of phosphorylation by BvgS, such as under modulating conditions. Interestingly, P2 is stronger in BPSMΔBvgA than in modulated BPSM, suggesting that nonphosphorylated BvgA may inhibit transcription driven by P2. However, we did not detect BvgA binding to the P2 region under modulating conditions, suggesting that nonphosphorylated BvgA does not bind to its own promoter region. Inhibition of P2 activity by nonphosphorylated BvgA may thus occur indirectly.

The ChIPseq analyses revealed 148 BvgA-binding sites in the BPSM chromosome. We arbitrarily grouped them into 3 categories depending on their localization with respect to the corresponding ORF, either located within putative promoter regions (category 1), within ORFs (category 2), or in noncoding regions (category 3). Some of the BvgA-binding sites are located in intergenic regions between two divergently transcribed genes. In this case, it is not possible to determine to which gene the BvgA-binding site corresponds. In some cases, the information may come from the RNAseq analyses if one of the two genes has been identified as a *vag*. However, even in this case, it cannot be excluded that the gene not identified as *vag* by RNAseq may under certain conditions also be regulated by BvgA. Conversely, when two *vag*s identified by RNAseq present a common promoter region, such as is the case for *bvgA/S* and *fhaB*, a unique BvgA-binding site can be detected that is a mean between the two or more actual BvgA-binding sites on the *bvgA* and *fhaB* promoter region.

In many cases, the BvgA-binding site could readily be located within the putative promoter region of *vag*s identified by the transcriptomic studies. However, several BvgA-binding sites were detected within ORFs, some of which were identified as *vag*s. ChIPseq studies in other bacteria have demonstrated that transcription factors may bind to sites within ORFs (16–19). Although the function of such binding is still largely unknown, it was recently shown that noncanonical binding within ORFs of several Escherichia coli genes may in fact drive transcription of highly unstable RNAs and may have an impact on genome evolution (16). It should be kept in mind that the Tohama I BX470248 genome annotation may present some errors, as exemplified by the recent correction of the *fmtB* translational start site (20), which was originally proposed to be 99 bp upstream of the actual experimentally determined initiation codon. A BvgA-binding site was detected by ChIPseq close to the 5′ end of *fmtB*, with a peak at position −71 from the experimentally determined translational initiation codon. This BvgA-binding site would have been positioned within the ORF centered at position +28 if the original annotation had been considered. In addition, the technology used here to sequence the DNA fragments isolated by ChIP does not allow us to determine the exact sequence to the nucleotide level to which BvgA binds. Hence, for some genes, in particular, those without a 5′ UTR, the BvgA-binding site detected by the ChIPseq analysis may overlap the ORF. Furthermore, it has been shown that several promoter regions of genes regulated by BvgA contain multiple BvgA-binding sites, some with high and some with low affinity (21–23). Therefore, the ChIPseq data define a zone were BvgA binds but do not identify the precise binding site to the nucleotide level.

Some *bona fide vag*s had no obvious BvgA-binding site. Some of them (*bp0499*, *bp0500*, *bp2232*, *bp2233*, *bp2256*, and *bp2257*) are members of the type III secretion system loci and are regulated by BrpL (8), the gene for which contains a strong BvgA-binding site in its putative promoter region and was shown to be directly regulated by BvgA (7). These genes are thus regulated by a regulation cascade involving BrpL whose expression is regulated directly by BvgA. Similar regulation cascades may also control the expression of other *vag*s lacking a BvgA-binding site. A total of 10 genes in that category were identified, including *fhaL* and *dnt*. The direct regulators of these genes remain to be identified. The *vag*s coding for regulatory proteins are obvious candidates.

Surprisingly, we found 10 BvgA-binding sites in proximity of *vrg*s. The expression of most *vrg*s depends on the transcription factor RisA (3), and 5 RisA-dependent *vrg*s contain BvgA-binding sites. These observations suggest that these genes are regulated by a dual mechanism: transactivation by RisA and repression by BvgA. Five of the *vrg*s containing BvgA-binding sites are not regulated by RisA. Their expression is enhanced in the absence of BvgA and under modulating conditions, even in the absence of RisA, suggesting that for these genes, phosphorylated BvgA acts as a repressor.

Among the 148 BvgA-binding sites, 91 are in proximity of genes that were not defined as *vags* or as *vrg*s by our RNAseq analysis. Hence, in addition to the RNAseq data shown here, we therefore also examined published data coming from other transcriptomic analyses of B. pertussis and Bordetella bronchiseptica (5, 7, 8, 24). One of them was identified as a *vag* by Moon et al. (7) and one as a *vrg*. The data set of Ahuja et al. (8) allowed us to identify 6 orthologue genes regulated as *vag*s and 4 orthologue genes regulated as *vrg*s. The data set of Gestal et al. (5) allowed us to identify an additional 25 genes that are overexpressed in the presence of serum or blood only in a BvgS-producing strain and not in a BvgS mutant but are not BvgS-regulated in the absence of blood or serum. All together, these investigations identified 31 different genes that were not found to be regulated in our RNAseq study but were shown to be regulated by BvgS under other conditions. Additionally, 4 of these 91 nonregulated genes were shown to be more expressed by the addition of blood or serum in the culture media but independently of the presence of BvgS, and 2 of these 91 nonregulated genes are more expressed *in vivo* in mice than *in vitro* (24). For the remaining 56 genes presenting BvgA-binding sites, it is unclear whether they are differentially regulated under conditions not examined so far, such as during the course of whooping cough in human patients, or whether they contain nonproductive BvgA-binding sites.

The analyses of the BvgA-binding site sequences in the putative promoter region allowed the MEME algorithm to identify a consensus motif similar to that already published (15), strengthening the motif already described and the detection method used in our study. However, when using the BvgA-binding site sequences found within ORFs, we were not able to identify a consensus motif. The binding sites found in the putative promoter regions are distributed randomly in the 201-bp regions used as the template (data not shown), probably because promoter regions of BvgA-regulated genes may contain from 1 to 10 binding sites with weak or strong affinity (21–23).

In conclusion, this combined RNAseq and ChIPseq study provides new insights in the BvgA-dependent regulation cascade, identifies bona fide directly BvgA-regulated genes, and uncovers surprising new features of the Bvg regulon, such as BvgA binding to *vrgs*. Therefore, these findings open new avenues for the study of the complex virulence regulation by B. pertussis.

## MATERIALS AND METHODS

### Construction of B. pertussis mutant strains.

The B. pertussis strains used in this study were derived from the Tohama I derivative BPSM (9). B. pertussis BPSMΔBvgA was obtained by homologous recombination using pSS1129 as an allelic exchange vector (25). The recombinant plasmids were introduced into B. pertussis by conjugation via Escherichia coli SM10 (26).

BPSMΔBvgA carries a 570-bp internal deletion in *bvgA* (*bp1878*) and was obtained as follows. Two 400-bp DNA fragments flanking the region to be deleted were obtained by PCR using the BPSM genomic DNA as the template and the oligonucleotide pairs 5′-AGACTTGAGAGCATCGCTACCATTCTAGATGAAATCCAGTGCCATAGTCT-3′ and 5′-TACAGGGTGATCGTCAATGATG-3′ and 5′-CATCATTGACGATCACCCTGTAAAACGCAACAATCTCGCCTAGC-3′ and 5′-ATAAGCTTGCCATTGACGGTGCCGATGAG-3′ as primers. The two resulting PCR products were used as the template with the first and last oligonucleotides described above to obtain an 865-bp final DNA fragment. The resulting XbaI-HindIII fragment was then introduced into XbaI-HindIII-digested pSS1129, yielding pSS1129ΔBvgA. This construct was used for allelic exchange in BPSM, yielding BPSMΔBvgA that produces a truncated form of BvgA composed of the 13 first and 6 last amino acids of BvgA.

### RNA extractions.

B. pertussis strains were grown on BG agar plates for 2 days at 37°C and then cultured in modified Stainer-Scholte (SS) liquid medium supplemented when indicated with 50 mM MgSO_4_ at 37°C under agitation. The bacterial cultures were stopped at mid-exponential phase (optical density at 600 nm [OD_600_] of 1.5 to 2) by adding 2 ml of a mixture of 5:95 phenol/ethanol (vol/vol) to 8 ml of bacterial suspensions. Bacteria were pelleted, and total RNA was extracted using TRI Reagent (Invitrogen) according to the manufacturer’s instructions. Genomic DNA was removed by DNase I treatment (Sigma-Aldrich).

### Illumina RNA sequencing.

RNAseq experiments were performed on two independent cultures for each condition. For each RNAseq sample, DNA-depleted total RNA was treated with the Ribo-Zero rRNA removal kit (Illumina) according to the manufacturer’s recommendations. The rRNA-depleted RNA was then used to build the Illumina library using the TruSeq RNA library preparation kit, followed by sequencing on an Illumina NextSeq 500 benchtop sequencer on SR150 high-output run mode. The RNAseq data of each sample were analyzed using both Rockhopper v2.0.3 and SPARTA with the default parameters to calculate the RPKM and *P* values for each coding sequence with B. pertussis Tohama I BX470248 genome annotation (27).

### ChIP analysis.

The B. pertussis ChIP protocol was adapted from that described by Solans et al. (28). B. pertussis strains were grown on Bordet-Gengou (BG) agar plates for 2 days at 37°C and then cultured in modified Stainer-Scholte (SS) liquid medium supplemented when indicated with 50 mM MgSO_4_ at 37°C under agitation. The bacterial cultures were stopped at mid-exponential phase (OD_600_ of 1.5 to 2) by adding 1% final concentration of formaldehyde and incubating for 30 min at 37°C with gentle shaking. Next, 125 mM glycine was added to saturate the cross-linking reaction and incubated for 30 min at 37°C with gentle shaking. The bacterial suspension was centrifuged at 5,000 × *g* for 10 min and washed twice with phosphate-buffered saline (PBS). Cell pellets were resuspended in immunoprecipitation buffer plus antiprotease Mini (Roche). Bacteria were lysed by sonication with a refrigerated Diagenode Bioruptor at 4°C with specific TPX Diagenode tubes. Cell debris was removed by centrifugation (15 min, 4,000 rpm, 4°C). To check the DNA fragmentation before immunoprecipitation (IP), a sample of DNA fragments was heated for 6 h at 65°C, treated with RNase A and proteinase K, and precipitated. The fragment size distribution was checked on a bioanalyzer to obtain a fragmentation size between 150 bp and 600 bp. IP was performed with anti-BvgA antibodies that recognize phosphorylated and nonphosphorylated BvgA equally well (29), and the samples were incubated at 4°C on a wheel for 16 h. Protein G magnetic Dynabeads (Invitrogen) were added to the IP samples and incubated for 4 h at 4°C on the wheel. Beads were separated from the lysate with a magnet and washed with IP buffer as in described in reference 28. Beads were finally resuspended in Tris-EDTA (TE) buffer and incubated for 6 h at 65°C. The supernatant was then treated with RNase A and proteinase K, and the DNA fragments were extracted with phenol-chloroform and precipitated with isopropanol. The ChIP procedure was performed on two independent cultures of each strain and on one culture of BPSM without BvgA antibody as a ChIP negative control.

### Illumina ChIPseq sequencing.

The DNA fragments isolated by ChIP were used to build the Illumina library using the Illumina TruSeq ChIP library preparation kit, followed by sequencing on an Illumina NextSeq 500 benchtop sequencer on SR150 high-output run mode. The ChIPseq data of each sample were analyzed using the ChIPseq analysis module of CLC Genomics v11.0 using the default parameters and the B. pertussis Tohama I BX470248 genome annotation to map reads, do the peak calling, and calculate peak shape scores. To avoid false positives and to increase clarity, ChIPseq peaks were considered informative if the CLC Genomics peak shape score was >5 and if the number of reads at the center of the peak was >1,000. The BvgA ChIPseq data obtained from the BPSM cultures were biologically reproducible between the two experiments with a correlation value *R***^2^** of 0.897 (data not shown). To determine the ChIPseq enriched regions, read alignments were analyzed using the peak caller software MACS2 version 2.1.2 with the following command: “macs2 callpeak -t CHIP_FILE –c CONTROL_FILE **–**format=BAM **–**name=bvgA **–**gsize = 2860332 **–**broad **–**nomodel -p 0.00001” (30). The mapped read depth was calculated on ChIPseq CLC Genomics output bam files using the SAMtools depth module of SAMtools (31).

### RACE analysis.

For each RACE sample, total RNA, extracted as described above, was treated with GeneRacer kit (Invitrogen) according to the manufacturer’s instructions and using BvgA RACE primer ATCAGGACCCGGACGGCGAATC. The cDNA isolated by the RACE experiment was then used to build the Illumina library using the Illumina TruSeq ChIP library preparation kit, followed by sequencing on an Illumina NextSeq 500 benchtop sequencer on SR150 low-output run mode.

### Quantitative real-time PCR.

Generation of cDNA and quantitative PCR (qPCR) were performed as described by Coutte et al. (3) using primers BvgA RACE (ATCAGGACCCGGACGGCGAATC), qPCR P1 BvgA (CACTCATGCCCGTATCGTTG), and qPCR P2 BvgA (AGCCATTCCTTTGACGCATC). At completion of the qPCR run, a dissociation curve from 55°C to 95°C was run to verify that a single product was generated. The efficiency of amplification for each primer pair was determined by serial dilution. The expression of the housekeeping gene *bp3416* was used as reference to normalize the data (3). The experiments were performed on two replicative cultures with at least 3 technical replicates for each condition.

### Data availability.

RNAseq data have been deposited at Gene Expression Omnibus under accession GSE137180, and ChIPseq data were deposited at Gene Expression Omnibus under accession GSE137027.

## Supplementary Material

Reviewer comments
