# Supplementary figures and images for: Combined RNAseq and ChIPseq Analyses of the BvgA Virulence Regulator of Bordetella pertussis
Source: mSystems. 2020 May 19;5(3):e00208-20. doi: 10.1128/mSystems.00208-20 (PMC7253368; doi:10.1128/mSystems.00208-20)

Figure S1

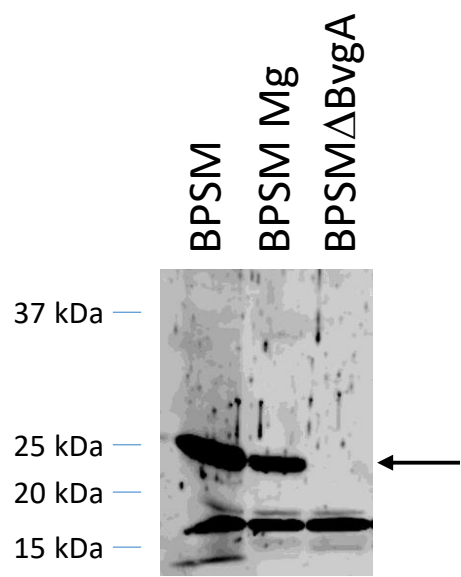

Supplement: FIG S1 [file mSystems.00208-20-sf001.pdf]

Figure S2

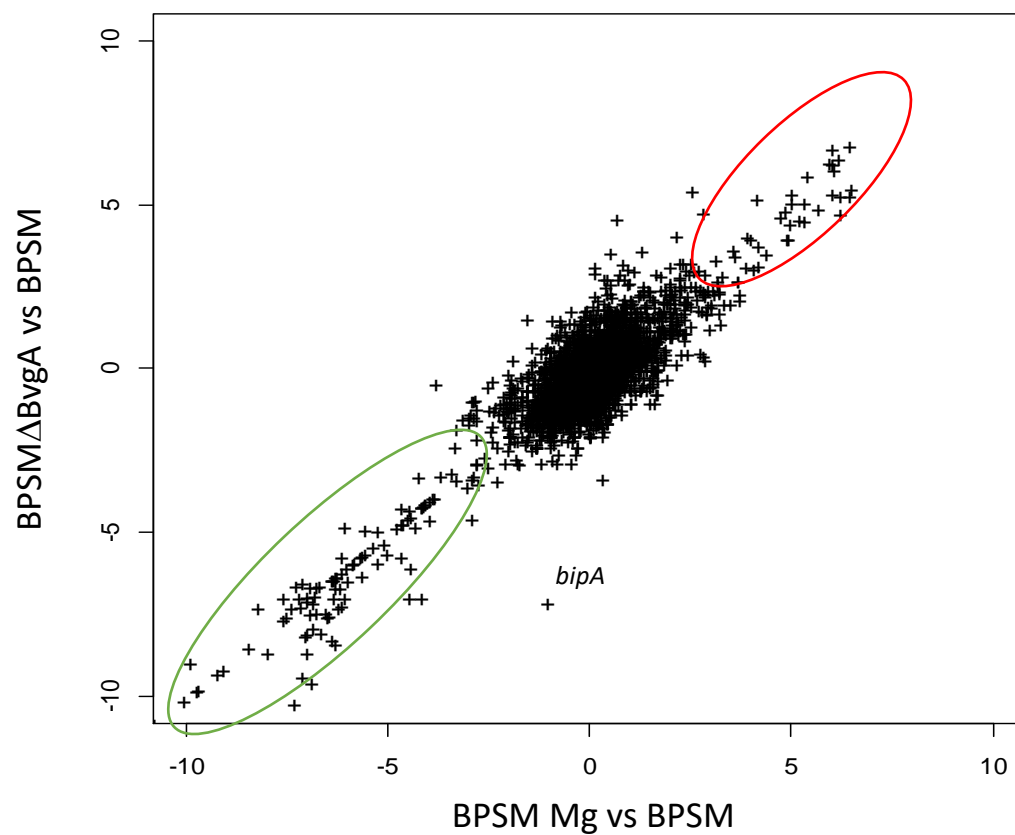

Supplement: FIG S2 [file mSystems.00208-20-sf002.pdf]

**Figure S3**

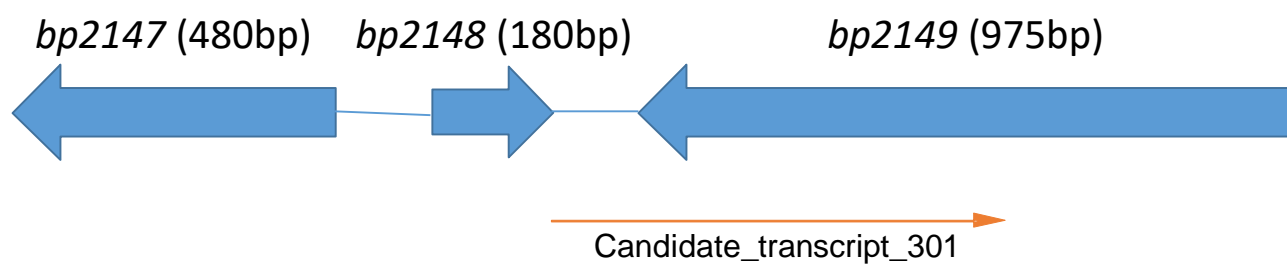

Supplement: FIG S3 [file mSystems.00208-20-sf003.pdf]

**Figure S4**

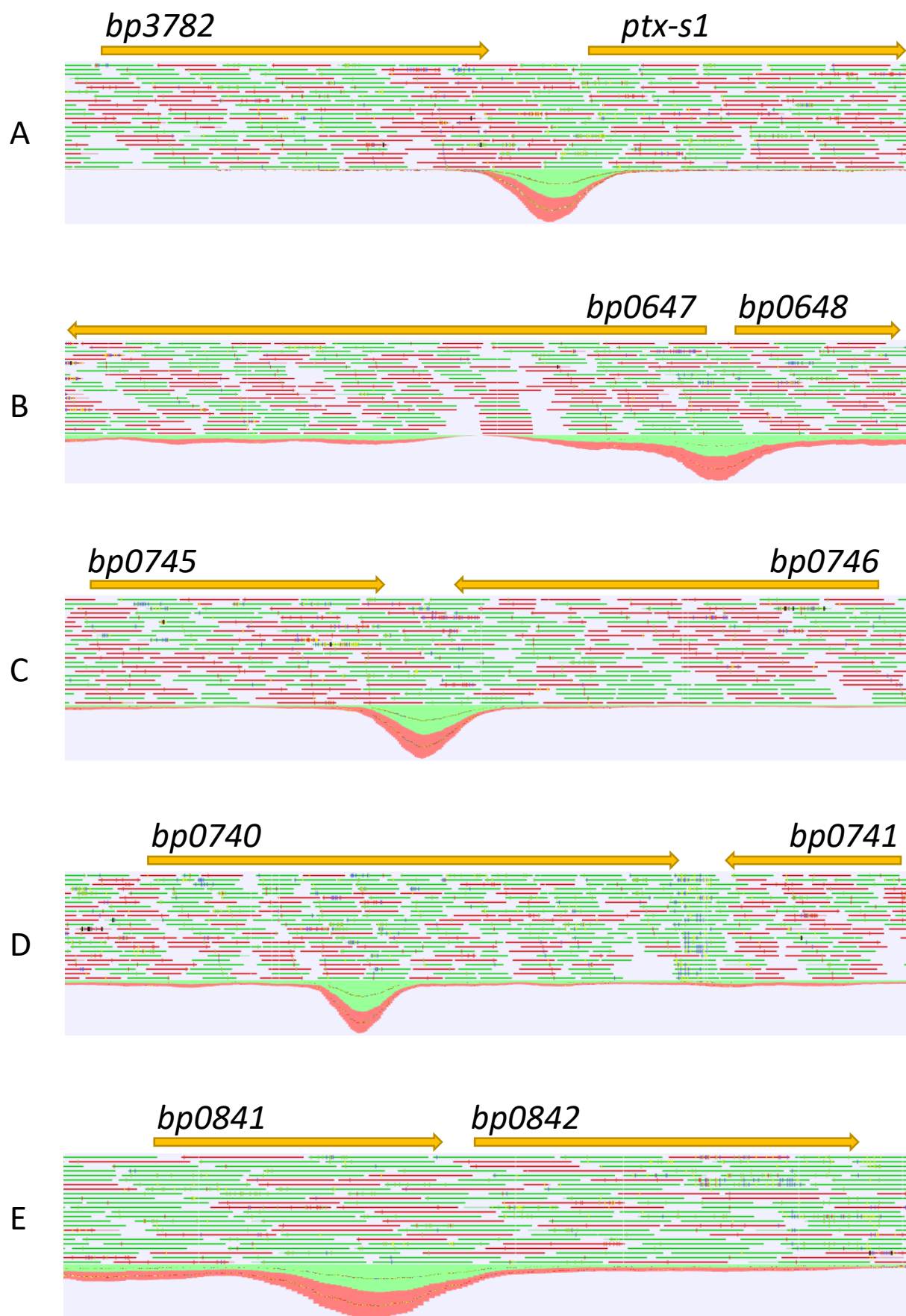

Supplement: FIG S4 [file mSystems.00208-20-sf004.pdf]

**Figure S5**

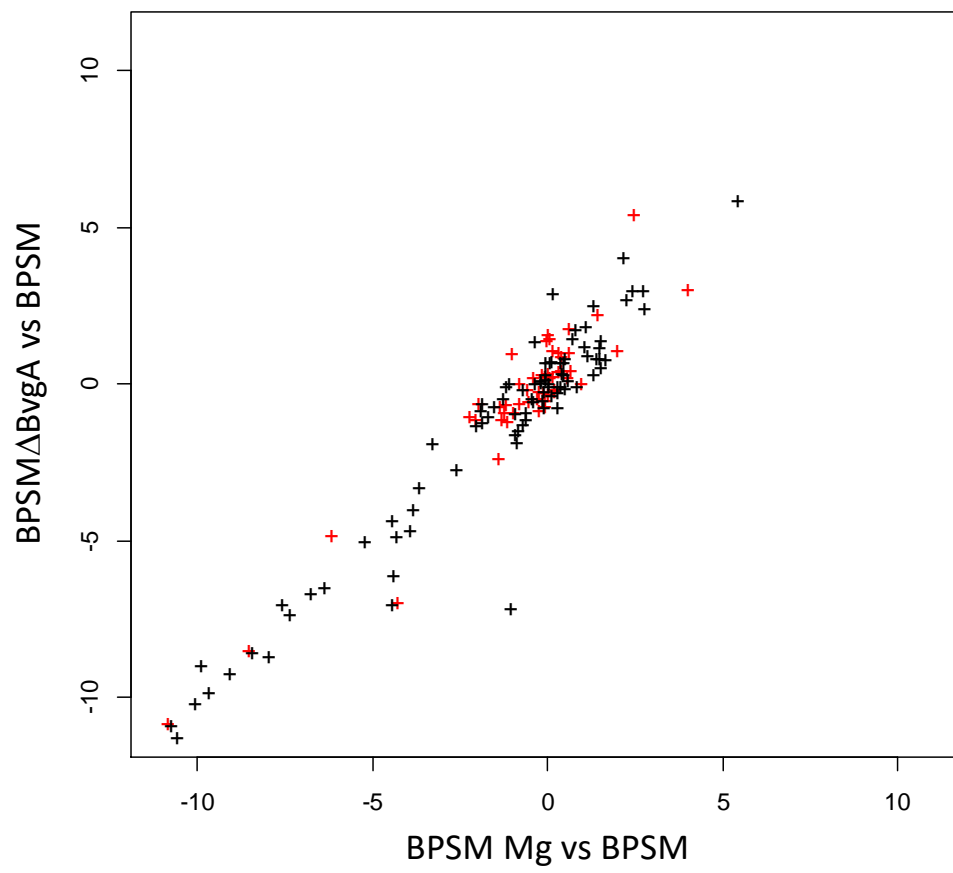

Supplement: FIG S5 [file mSystems.00208-20-sf005.pdf]

Figure S6

A

*fim2*

*maeB*

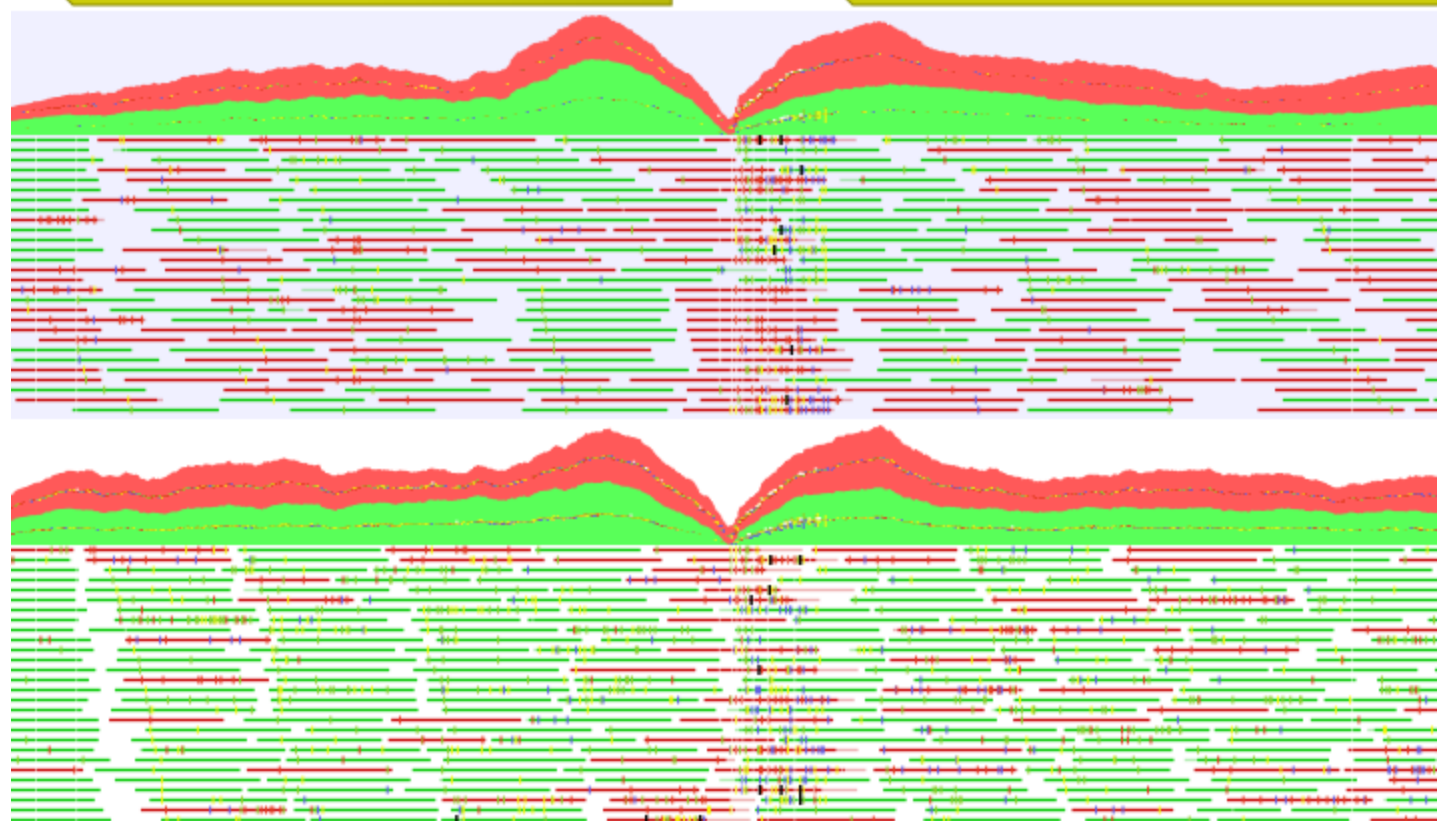

B

*bp1567*

*fim3*

*bp1569*

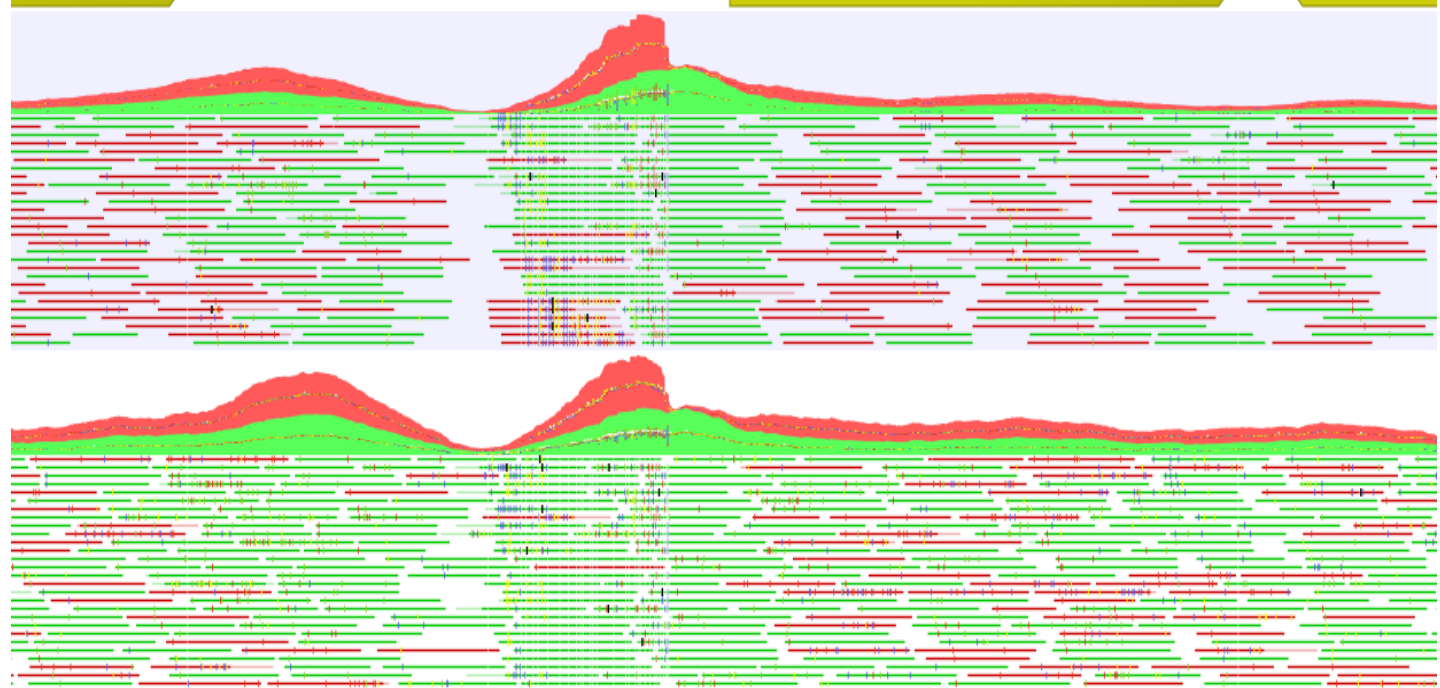

Supplement: FIG S6 [file mSystems.00208-20-sf006.pdf]

A

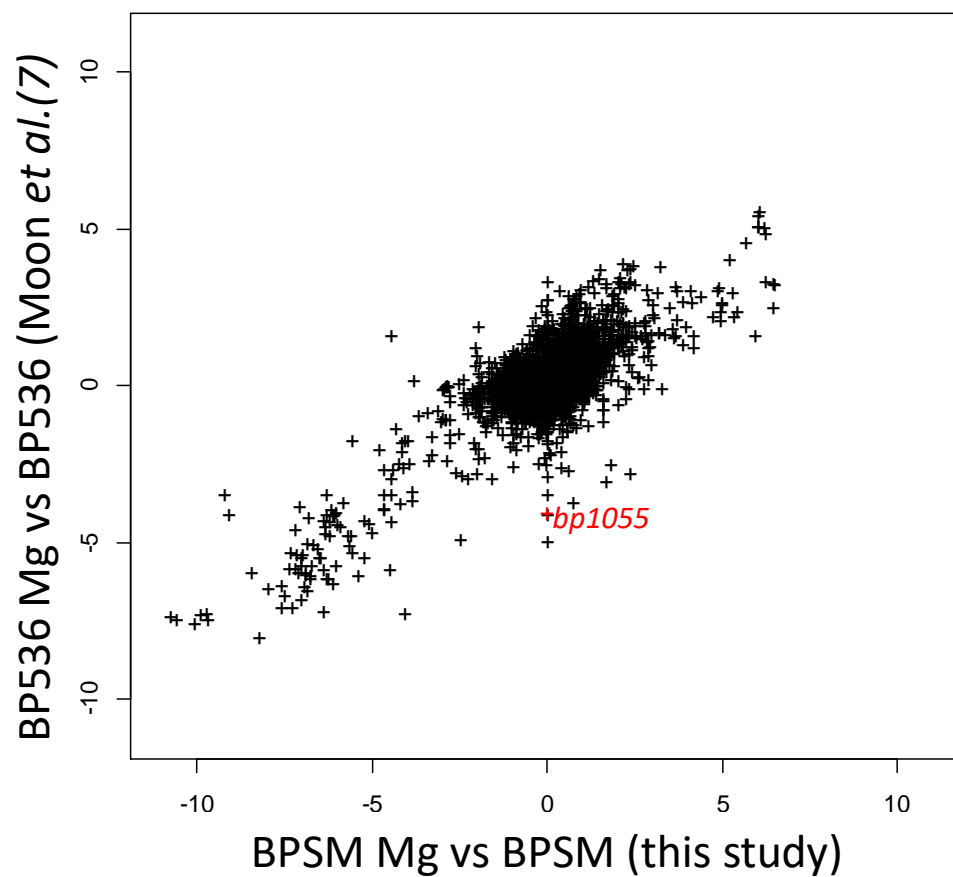

B

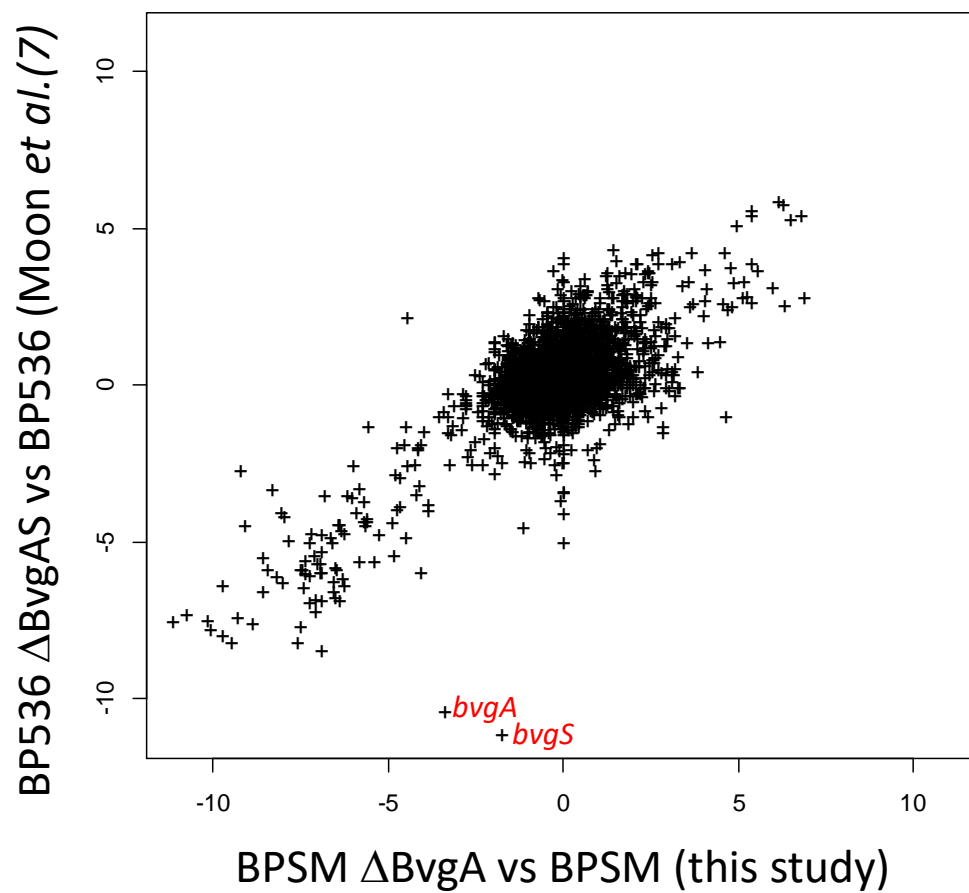

Supplement: FIG S8 [file mSystems.00208-20-sf008.pdf]
