# Supplementary material for: Combined RNAseq and ChIPseq Analyses of the BvgA Virulence Regulator of Bordetella pertussis
Source: mSystems. 2020 May 19;5(3):e00208-20. doi: 10.1128/mSystems.00208-20 (PMC7253368; doi:10.1128/mSystems.00208-20)
Supplement: FIG S7 [file mSystems.00208-20-sf007.pdf]

Figure S7

gene	Product	Synonym	BPSM Mg vs BPSM	BPSMΔBvgA vs BPSM
bp0022	Two-component response regulator		-0.47	-0.26
bp0142	GntR family transcriptional regulator		-1.51	-1.15
bp0764	probable LysR-family transcriptional regulator	*cyaX*	-4.17	-4.17
bp1021	RNA polymerase sigma factor for flagellar operon	*flaD*	0.00	0.00
bp1417	[protein-PII] uridylyltransferase	*glnD*	-0.28	-1.06
bp1496	probable two-component response regulator		-4.48	-4.26
bp1876	regulatory protein BvgR	*bvgR*	-6.38	-6.39
bp1877	virulence sensor protein	*bvgS*	-2.02	-1.78
bp1878	virulence factors transcription regulator	*bvgA*	-2.29	-3.39
bp2234	putative RNA polymerase sigma factor	*brpL*	-8.00	-8.59
bp2268	methyl-accepting chemotaxis protein		0.00	0.00
bp2399	putative transcriptional regulator		-0.14	-0.64
bp2507	ferric ion uptake regulator	*fur*	0.14	1.51
bp2520	putative LysR-family regulatory protein		-0.19	1.14
bp3350	two component response regulator		0.41	-0.20

Log_2FC

-7 -6 -5 -4 -3 -2 -1 0 1 2 3 4 5 6 7
