# Supplementary material for: Combined RNAseq and ChIPseq Analyses of the BvgA Virulence Regulator of Bordetella pertussis
Source: mSystems. 2020 May 19;5(3):e00208-20. doi: 10.1128/mSystems.00208-20 (PMC7253368; doi:10.1128/mSystems.00208-20)
Supplement: Reviewer comments [file mSystems.00208-20_review_history.pdf]

AMERICAN SOCIETY FOR MICROBIOLOGY | mSystems®

Combined RNAseq and ChIPseq analyses of the BvgA virulence regulator of Bordetella pertussis

Loic Coutte, Rudy Antoine, Stephanie slupeck, Luis Solans, Julien Derop, Amelie Bonnefond, David Hot, and Camille Locht

Corresponding Author(s): Loic Coutte, INSERM

Review Timeline:

Submission Date:	March 10, 2020
Editorial Decision:	April 8, 2020
Revision Received:	April 20, 2020
Accepted:	April 24, 2020

Editor: Mark Mandel

Reviewer(s): The reviewers have opted to remain anonymous.

Transaction Report:

(Note: With the exception of the correction of typographical or spelling errors that could be a source of ambiguity, letters and reports are not edited. The original formatting of letters and referee reports may not be reflected in this compilation.)

DOI: https://doi.org/10.1128/mSystems.00208-20

Reviewer comments:

Reviewer #1 (Comments for the Author):

This manuscript describes the identification of the BvgA regulon in Bordetella pertussis using a combination of RNA-seq and ChIP-seq. Previous studies have identified BvgA-regulated genes using microarray analysis of RNA levels or RNA-seq. Without having complementary information on BvgA binding sites, it is not possible to use these data alone to identify directly regulated genes. Hence, the current study is an important advance beyond previous work. Overall, I think the data are sound, and provide valuable information that warrant publication in mSystems. Nonetheless, the rigor of the data analysis, and the data presentation can be improved considerably. I also recommend greatly shortening the paper, distilling it down to the key result: identification of directly and indirectly BvgA-regulated genes.

We have shortened the paper as much as we could, following the precise suggestions made by the referee below.

Major comments:

- The description of highly expressed and weakly expressed genes (independent of BvgA) is tangential to the theme of the paper. I recommend removing this text and table, and the first page of the discussion.
As requested by the referee, we have modified the paragraphs on the BvgA-independent highly and weakly expressed genes in the results and discussion sections and have removed Table2 in the revised version. See page 5, 6 and 15 of the marked-up copy of the manuscript.

- The authors should rigorously compare their RNA-seq data to those from previous studies. For example, a figure like Figure S2 would be useful to compare to the Moon et al. dataset.
Following the request of the referee, we have made a supplementary Figure S7 to illustrate the comparison between the Moon et al. and Coutte et al. RNAseq data in the discussion section. See page 16 of the marked-up copy of the manuscript

- Page 6. The strategy to identify BvgA-regulated genes from RNA-seq data uses only fold change information. It would be good to incorporate a more rigorous statistical test to account for variability between replicates. There are lots of tools available for this type of analysis, e.g. Rockhopper, DEseq.
In fact, the analysis was done with Rockhopper and the qValues were included in each table (Table 3, Tables S1 to S4). The Rockhopper analysis was done with the two sets of data for each RNAseq condition: 2 for BPSM, 2 for BPSM MG and 2 for BPSM ΔBvgA. All the data sets were analyzed altogether in a same Rockhopper analysis using the default parameter. The obtained results provided the calculated RPKM in each condition, the RPKM ratios between conditions and the calculated qValues presented in Table 3 and Tables S1, S2 & S4. We also run SPARTA that uses the DEseq algorithm on our RNAseq data. The SPARTA results Log_2FC and Pvalues are now also included in Table 3 and Tables S1, S2 & S4. The strategy to use fold change was done to avoid over interpretation of the data and to focus on genes strongly up- or down-regulated, hence the fold change cut off used of ±2 gave us 365 regulated genes with SPARTA calculated Pvalues between $4.3x10^{-270}$ and 0.051. See page 7 of the marked-up copy of the manuscript and Table 3 and Tables S1, S2 & S4.

- Page 8. The sRNA predictions are interesting, but require follow-up work to be convincing,

e.g. a Northern blot +/- bvgA. Perhaps this can be done for the putative sRNA with an upstream BvgA binding site.
We identified only two sRNA with an upstream BvgA-binding site. One was found upstream of fim3, called vrgX and the RNA transcript was already shown by northern blot analysis in Chen et al. 2018. Mol. Microbiol (13). The other BvgA-binding site was found close to the putative novel sRNA upstream of bp0258. Indeed, additional Northern blot experiments may be required to confirm this putative sRNA. However, we feel that sRNA characterization is not the main aim of this manuscript, which has to be shorted as requested by the reviewers. None of the other sRNA identified by RNAseq presented a BvgA-binding site.

- It is difficult to assess the quality of the ChIP-seq data and the analysis of these data. The parameters used by the peak caller seem to be arbitrary. I suggest using an established peak-calling algorithm such as MACS.
The CLC genomics peak caller was used with default parameters, resulting in the identification of 2055 peaks with peak shape scores comprised between 1.28 and 29.15 and with associated Pvalues comprised between 3.64×10^{-187} and 0.1. The information corresponding to these 2055 detected peaks has now been added in table S6 to present all the data obtained by the peak caller. To avoid over interpretation of these data we chose to select peaks having a minimum peak shape score of 5 and a minimum mapped read at the center of the peak of 1000 (maximum reads observed at a peak is 24661). These thresholds result in a final selection of 148 peaks with peak shape scores comprised between 5 and 29.15 and with associated Pvalues comprised between 3.64×10^{-187} and 3.79×10^{-43}. See table S6

Reviewer # 1 suggests to use the peak-calling algorithm MACS. We have chosen to use the CLC genomics peak caller, as it was already used and published for ChIPseq analyses (e.g. Kazi et al. 2016. PLoS Pathog. 12:e1005570). Furthermore, a comparison between CLC peak caller and other peak callers, such as CisGenome, HOMER, and MACS did not show strong differences (Strino et al. 2016. BMC Bioinformatics 17 Suppl 5:206). Nevertheless, we run our data set BPSM run1 vs BPSM BvgA run 1 using MACS2. The MACS2 analysis using a Pvalue threshold of 1×10^{-5} gave us 143 detected peaks. The comparison of the MACS2 with the CLC analysis is represented in a figure only for the reviewer. Most of the 148 selected peaks found with CLC were also found by MACS2 with exception of 5 peaks located within an Insertion Sequences that have been deliberately excluded in the CLC peak caller analysis. The presented results show that the analysis using the CLC genomic peak caller is robust compared to MACS. see Figure for reviewers 1.

- The comparison of ChIP-seq and RNA-seq data needs to be more rigorous. First, I recommend presenting scatterplots of the RNA seq data (wt vs mutant, induced vs uninduced), highlighting genes immediately downstream of a ChIP-seq peak, and genes with an internal ChIP-seq peak. Second, there needs to be a systematic effort to identify genes that are differentially expressed and have an upstream ChIP-seq peak. This could include previously reported sRNAs.
As requested by the reviewer we made a scatterplot representation of the regulation of the genes showing a BvgA-binding site in the promoter region or within CDS in figure S6. As described in the Discussion section, we have not only compared the ChIP-seq analysis with our own RNAseq data, but also with other data available in the literature. See page 13 and 19 of the marked-up copy of the manuscript and figure S6.

- I extracted 101 nt regions around the ChIP-seq peaks, merging overlapping regions, and used MEME to search for an enriched motif. I found a strongly enriched motif that looks

similar to previously reported binding sites. Hence, I recommend trying the MEME analysis again. The default parameters on the MEME webserver are probably the best option. Assuming the authors can find an enriched motif, it would be useful to determine the position of putative binding sites relative to ChIP-seq peak centers. This can be done easily using the Centrimo tool on the MEME suite webserver.
As requested by the reviewer we have now done the MEME analysis again using the default parameters on the MEME webserver. The obtained results are presented in Figure 6.
We tried to detect a consensus sequence using 3 sets of data: all 148 BvgA-binding sites, the 91 BvgA-binding sites located in putative promoter regions and the 52 BvgA-binding sites located within ORFs.

Using all 148 BvgA-binding sites MEME found a 10 nucleotides motif enriched 70 times with an E-value of $6.8e^{-14}$.
Using the 91 BvgA-binding sites located within putative promoter regions MEME found a 10 nucleotides motif with an E-value of $2.0e^{-10}$ *enriched 64 times, and the motif wassimilar to that found using all 148 BvgA-binding sites.*
Using the 52 BvgA-binding sites located within CDS, MEME found a 11 nucleotides motif enriched 22 times with an E-value of $3.0e^{-2}$. *This motif is slightly different from those found in the 148 and 91 data sets. However, among these 52 BvgA-binding sites, 14 are located within a CDS close to the 5' end of an adjacent CDS and could therefore be part of the promoter region of the downstream gene (e.g. Fig. S4 E).*

The main problem to determine the precise position of BvgA-binding sites relative to ChIP-seq peak centers is that multiple BvgA-binding sites may be found in promoter regions of regulated genes. Thus, the ChIPseq peaks may represent the region of BvgA fixation covering several sites, and therefore the center of the peak represents the center of the region of fixation and not directly the precise region of binding.

See page 14and 20 of the marked-up copy of the manuscript and Figure 6

Additional comments:

- Page 5. The number of non-expressed genes varying between strains could simply be due to sequence read coverage differences between experiments. I recommend removing this text. `
As requested by the referee, we have removed the corresponding paragraph in the results and discussion sections. See page 5 and 15 of the marked-up copy of the manuscript

- Figure 1. The Venn diagrams should be drawn to scale.
The Venn diagrams have now been redrawn.

- Page 9. Speculation on the role of BvgA regulating other regulators is too preliminary.
We are not sure what the referee meant by this statement.

- Figure 3. The way the data are presented is confusing. For panel A, I suggest a scatterplot with distance from the start codon on the x-axis and number of sequenced 5' ends on the y-axis.
For panel A it is not possible to make a scatterplot because it is not possible to have a normalized quantification of the number of reads corresponding to the sequenced 5' ends, as there is no internal reference to normalize the sequencing data between samples, although the

different libraries were loaded at equimolarity. That is why we performed the qRT-PCR analysis presented in panel B.

For panel B, I get the basic idea, but I've never seen rtPCR data represented this way. Relative expression level, normalized to a control gene, would be a much simpler way to plot these data. More generally, I'm not sure how useful this experiment is, given that potential complications associated with deleting bvgA and the effect on RNA stability.
Panel B represents the strengths of the different bvgA promoters relative to the expression of the housekeeping gene bp3416 used as reference to normalize the data. We feel that these results are useful, as they allow the relative quantification of the bvgA promoter strength that cannot be done with only the RACE data presented in panel A.

- When discussing the location of BvgA binding sites, the authors should avoid the use of "promoter" since this has a very defined meaning. Instead, I suggest "upstream intergenic region".
Instead of "upstream intergenic region" we prefer to use "putative promoter region", as BvgA binding has already been published to occur in confirmed promoter regions of BvgA-regulated genes. Since indeed the ChIPseq results shown here cannot be taken as prove of BvgA binding in promoters in all cases, we nevertheless prefer the term "putative promoter region".

- Figure S4. What are the green and red graphs? If these show data for forward and reverse strand reads, the authors should explain why the peak heights differ for each. What are the lines within the red and green graphs? The details of this figure panel should be explained more clearly in the legend.
Single reads mapping in their forward direction are in green. Single reads mapping in their reverse direction are red. Any mismatches between the read and reference are shown as colored dots. More reads are mapped to the reference than can be shown in detail. Therefore, these reads are displayed in an overflow graph below the reads. The overflow graph is shown in the same colors as the reads, and mismatches in reads are shown as colored narrow vertical lines within the overflow graph. This description has now been added in the figure legends.

- Figure 5. This figure adds very little to the paper. Intergenic binding sites are expected to be close to gene starts, and there is no obvious pattern for the intragenic sites.
As requested by the referee, figure 5 has now been removed and the references to the data presented in this figure were notified as "data not shown" in the manuscript text.

- Page 12. The text refers to bioinformatic data processing that removes stretches of cytosines from reads. No details are given for this processing. If it complicates the analysis, I suggest removing this step.
This bioinformatic data processing cannot be removed, as it is a part of the Illumina base calling data processing directly from the sequencer that excludes reads beginning with stretches of cytosines considering them as sequencing artefacts.

- Page 12. What is an "atypical" ChIP-seq peak? It is not clear why this would clearly be a binding site.
We add a supplementary figure to show the atypical ChIP-seq peak close to fim2 and fim3 as Figure S5

- Page 17. The authors refer to a recent paper on FliA that shows FliA-dependent transcription of many intragenic RNAs in Escherichia coli. It is important to note that FliA is a Sigma factor. Hence, I don't think this is a relevant comparison.
We agree with the reviewer and removed reference 22. See page 27 of the marked-up copy of the manuscript

- Page 18. Transcription factor binding sites that are not associated with detectable regulation are fairly common. See these reviews: PMID 22983621 and PMID 17581117.
We thank the reviewer for the suggestion and have added the suggested references. See page 18 and 27 of the marked-up copy of the manuscript

Reviewer #2 (Comments for the Author):

This paper reports RNA-seq and ChIP-seq results for the B. pertussis global regulator of virulence BvgA. The authors use 3 conditions: wt, delete BvgA, and modulation (growth in the presence of MgSO4 which is known to eliminate BvgA phosphorylation by its cognate sensor kinase BvgS). The authors compare the results of their study to previous RNA-seq analyses of the BvgA regulon for B. pertussis grown with and without MgSO4 or in the mouse respiratory tract.

Although previous transcriptomic analyses of modulated/unmodulated B. pertussis have been reported, this paper is useful to the field as confirmation of the earlier work and by providing ChIP-seq analyses to determine the binding sites for BvgA. My comments are listed below.

p. 6 and p.11 Given that bipA can be activated or repressed depending on the level of BvgA~P, I don't think that it is correct to surmise that it is not affected by modulation. Rather it is not affected under these particular conditions.
In agreement with the referee, the text has been changed. See page 6 and 12 of the marked-up copy of the manuscript

p. 16 - The high level of BvgA under modulating conditions has previously been demonstrated. Please reference Boulanger et al. 2013 Mol Micro
This reference has now been added. See page 27 of the marked-up copy of the manuscript

p. 18 - Direct activation of the BrpL promoter has previously been shown (See Moon et al 2017).
The text has been changed. See page 19 of the marked-up copy of the manuscript

Please indicate the cut-off values for the q-value scores used as a criteria.
As explained in the reply to the comments of reviewer # 1 to avoid over interpretation of the ChIPseq data we chose to select peaks having a minimum peak shape score of 5 and a minimum mapped read at the center of the peak of 1000. These thresholds result in a final selection of 148 peaks with peak shape score comprised between 5 and 29.15 and with associated Pvalues comprised between $3.64x10^{-187}$ and $3.79x10^{-43}$. . See page 7 of the marked-up copy of the manuscript and Table 3 and Tables S1, S2 & S4

Please provide titles and legends for the Tables and the Supplemental Tables.
Titles are now provided for all the tables

I think the paper can be shortened somewhat by not repeating results in the Discussion section. I suggest the authors read carefully to remove as much repetition with the Results as possible.

We have removed repetitions as much as possible.

Also Fig. 4 is not necessary.

Fig4 has been removed

April 8, 2020

Dr. Loic Coutte
INSERM
microbiology
U1019-CIIL-Institut Pasteur de Lille
1 rue du Pr Calmette
Lille 59021
France

Re: mSystems00208-20 (Combined RNAseq and ChIPseq analyses of the BvgA virulence regulator of Bordetella pertussis)

Dear Dr. Loic Coutte:

Thank you for submitting your manuscript to mSystems. I sent the manuscript to two experts in the field, and below you will find the comments of the reviewers. These are the same reviewers as the previous submission. It is clear that the revisions made have substantially improved the manuscript. Both reviewers had minor comments that need to be addressed prior to acceptance of the manuscript. In particular, please address Reviewer 1's question about peak-calling and MAC2 versus CLC Genomics Workbench. I share the reviewer's concern -- CLC's closed-source nature makes it difficult to assess its methods. The paper you cited in your response to the authors is written by two employees of Qiagen, the company that now produces the software. It seems from your reply to authors that you may already have the relevant analysis, but it should be presented and discussed in the manuscript. It is my expectation that this comment and the others can be addressed without the need for further wet-lab experimentation.

To submit your modified manuscript, log onto the eJP submission site at https://msystems.msubmit.net/cgi-bin/main.plex. If you cannot remember your password, click the "Can't remember your password?" link and follow the instructions on the screen. Go to Author Tasks and click the appropriate manuscript title to begin the resubmission process. The information that you entered when you first submitted the paper will be displayed. Please update the information as necessary. Provide (1) point-by-point responses to the issues raised by the reviewers as file type "Response to Reviewers," not in your cover letter, and (2) a PDF file that indicates the changes from the original submission (by highlighting or underlining the changes) as file type "Marked Up Manuscript - For Review Only."

Due to the SARS-CoV-2 pandemic, our typical 60 day deadline for revisions will not be applied. I hope that you will be able to submit a revised manuscript soon, but want to reassure you that the journal will be flexible in terms of timing, particularly if experimental revisions are needed. When you are ready to resubmit, please know that our staff and Editors are working remotely and handling submissions without delay. If you do not wish to modify the manuscript and prefer to submit it to another journal, please notify me of your decision immediately so that the manuscript may be formally withdrawn from consideration by mSystems.

To avoid unnecessary delay in publication should your modified manuscript be accepted, it is important that all elements you upload meet the technical requirements for production. I strongly

recommend that you check your digital images using the Rapid Inspector tool at http://rapidinspector.cadmus.com/RapidInspector/zmw/.

If your manuscript is accepted for publication, you will be contacted separately about payment when the proofs are issued; please follow the instructions in that e-mail. Arrangements for payment must be made before your article is published. For a complete list of **Publication Fees**, including supplemental material costs, please visit our website.

Corresponding authors may join or renew ASM membership to obtain discounts on publication fees. Need to upgrade your membership level? Please contact Customer Service at Service@asmusa.org.

Thank you for submitting your paper to mSystems.

Sincerely,

Mark Mandel

Editor, mSystems

Journals Department
American Society for Microbiology
1752 N St., NW
Washington, DC 20036
E-mail: peerreview@asmusa.org
Phone: 1-202-942-9338

Reviewer comments:

Reviewer #1 (Comments for the Author):

The authors have made extensive changes to the manuscript, which is now much more compact and easier to read. I have only minor concerns:

- Figures 2 and 3 are thematically linked and should be merged

- I still don't understand why the 5' RACE data in Figure 3A show reads on both strands.

- Figure 3B is insufficiently described in the legend and in the figure itself.

- "Putative promoter region" is too vague. The authors should define it, e.g. intergenic region upstream of a gene, or within set distances either side of a gene start.

- The p-values for ChIP-seq peak calling are clearly meaningless. I still think it makes more sense to use a more established method like MACS2. How do the MACS2 peaks compare to those identified in CLC Genomics Workbench? The figure for the reviewers doesn't really address this. How many peaks are shared in the two lists? If there are substantial differences in the lists, how does the presence/absence of the motif compare for peaks unique to each list?

- Figure S6A + B should be combined into a single plot that shows all genes, with the relevant genes highlighted in different colors.

- I don't understand the term "fixation site". It should either be defined or replaced with a more standard term.

- Figure S5. The odd nature of these ChIP-seq peaks could be due to phase-variable recombination in these regions, or due to high similarity between multiple regions that leads to incorrect read alignment. I wouldn't say these regions are "clearly" binding sites, but I think it's fair to say that the level of enrichment is consistent with them having binding sites.

- Figure 5 is very speculative and would be better in the supplement. Also, the numbers in some of the cells are too dark to read.

- E-values and the number of sites contributing to a motif should be included in Figure 6.

- The fact that the intragenic sites are much less strongly associated with a motif suggests that these are not genuine binding sites. Were these sites also found with MACS2? I disagree with the argument in the discussion that sites within ORFs might represent a different mode of binding - this is too strong a claim to make without additional supporting data.

- "consensual motif" should be "consensus motif".

- Line 396, "insides" should be "insights".

Reviewer #2 (Comments for the Author):

The authors have answered all of my concerns. Just one minor mistake:

line 357 please correct reference. It says #8, should be #7

Re: mSystems00208-20 (Combined RNAseq and ChIPseq analyses of the BvgA virulence regulator of Bordetella pertussis)

Dear Dr. Loic Coutte:

Thank you for submitting your manuscript to mSystems. I sent the manuscript to two experts in the field, and below you will find the comments of the reviewers. These are the same reviewers as the previous submission. It is clear that the revisions made have substantially improved the manuscript. Both reviewers had minor comments that need to be addressed prior to acceptance of the manuscript. In particular, please address Reviewer 1's question about peak-calling and MAC2 versus CLC Genomics Workbench. I share the reviewer's concern -- CLC's closed-source nature makes it difficult to assess its methods. The paper you cited in your response to the authors is written by two employees of Qiagen, the company that now produces the software. It seems from your reply to authors that you may already have the relevant analysis, but it should be presented and discussed in the manuscript. It is my expectation that this comment and the others can be addressed without the need for further wet-lab experimentation.

Response to editor comments:

Indeed, the given reference comparing CLC peak caller and MACS was written by employees of Qiagen. However, CLC genomics peak caller has also been used in many other published studies, not including employees of Qiagen. Here are some of them:
Kazi et al. PLoS Pathog. 2016;12:e1005570,
Polman et al. BMC Neurosci. 2012;13:118. doi:10.1186/1471-2202-13-118
Kratochwil et al. Mol Ecol Resour. 2015;15(4):761–771. doi:10.1111/1755-0998.12350
Perduns R et al. Plant Physiol. 2015;168(4):1378–1388. doi:10.1104/pp.15.00934
Antipov SS et al. PLoS One. 2017;12(8):e0182800. doi:10.1371/journal.pone.0182800

As requested we did indeed a pairwise analysis of BPSM with BPSMΔBvgA using MACS2 and found 143 peaks identified by MACS2 were all also found by CLC using the threshold value that we have chosen. Only three additional peaks were identified by CLC and they correspond to peaks located within an Insertion Sequences that have been deliberately excluded in the MACS2 peak caller analysis. More details are provided in the reply to referee #1. We have added the MACS2 list in the supplementary material of the revised version and added a sentence in that respect in lines 226 to 230 of the revised manuscript. We hope that this is acceptable.

Reviewer comments:

Reviewer #1 (Comments for the Author):

- Figures 2 and 3 are thematically linked and should be merged
Figures 2 and 3 have now been merged, and the manuscript has been modified accordingly.

- I still don't understand why the 5' RACE data in Figure 3A show reads on both strands.

For the RACE experiment cDNA was used to build the sequencing library using the Illumina TruSeq ChIP Library Preparation Kit, as specified in the M&M section. This TruSeq ChIP Library Preparation Kit is not a "stranded" Library Preparation Kit and does therefore not produce an orientated library in contrast to the TruSeq stranded RNA Library Preparation Kit used for the RNAseq experiments. Therefore, 5' RACE data show reads on both strands.

- Figure 3B is insufficiently described in the legend and in the figure itself.

The legend of Figure 3B, now Figure 2D, has been updated to add more details in order to increase the understanding of the presented data.

- "Putative promoter region" is too vague. The authors should define it, e.g. intergenic region upstream of a gene, or within set distances either side of a gene start.

We kept the term "Putative promoter region" but describe it in the manuscript as corresponding to a region upstream of an ORF within set distances between -5 and -806 nt to the ATG translational start codon and not overlapping with an adjacent ORF. (see lines 216 to 218 in the revised version)

- The p-values for ChIP-seq peak calling are clearly meaningless. I still think it makes more sense to use a more established method like MACS2. How do the MACS2 peaks compare to those identified in CLC Genomics Workbench? The figure for the reviewers doesn't really address this. How many peaks are shared in the two lists? If there are substantial differences in the lists, how does the presence/absence of the motif compare for peaks unique to each list?

We have indeed run our data set BPSM run1 vs BPSMΔBvgA run 1 using MACS2 genomics peak caller.
With a Pvalue threshold of $1.00e^{-2}$ MACS2 identified 174 peaks, while using a Pvalue threshold of $1.00e^{-5}$ it identified 143 peaks.
CLC peak caller identified 441 peaks with minimal Pvalue of $2.69e^{-10}$.
This list of 441 peaks identified by CLC dropped down to 146 peaks using the threshold of minimum 1000 reads at the center of the detected peaks to avoid miss interpretation due to weak sequencing depth.
The 143 peaks identified by MACS2 are all present in the list of the final 146 peaks identified by CLC. The additional peaks identified by CLC correspond to peaks located within Insertion Sequences that have been deliberately excluded in the MACS2 peak caller analysis. The comparison is now presented in a table in supplementary material and briefly commented on in lines 226 to 230 of the revised version of the manuscript, as well as added a few lines in the Methods section, lines 480-484.

Thus, all the peaks are present in both lists.

- Figure S6A + B should be combined into a single plot that shows all genes, with the relevant genes highlighted in different colors.

As requested by the reviewer Figures S6A+B have been merged, showing the data on a single plot.

- I don't understand the term "fixation site". It should either be defined or replaced with a more standard term.
The term "fixation site" was replaced in the revised manuscript by the more standard term "binding site"

- Figure S5. The odd nature of these ChIP-seq peaks could be due to phase-variable recombination in these regions, or due to high similarity between multiple regions that leads to incorrect read alignment. I wouldn't say these regions are "clearly" binding sites, but I think it's fair to say that the level of enrichment is consistent with them having binding sites.
The revised manuscript has been modified according to the reviewer's suggestion.

- Figure 5 is very speculative and would be better in the supplement. Also, the numbers in some of the cells are too dark to read.
The Figure has been modified and added to the supplementary materials as Figure S7. The numbers are now shown in white

- E-values and the number of sites contributing to a motif should be included in Figure 6.
The Figure has now been modified to include the E-values and the number of sites.

- The fact that the intragenic sites are much less strongly associated with a motif suggests that these are not genuine binding sites. Were these sites also found with MACS2? I disagree with the argument in the discussion that sites within ORFs might represent a different mode of binding - this is too strong a claim to make without additional supporting data.
As explained above, yes, all these sites were also found with MACS2. In the revised version we have deleted the statement about a potential different mode of BvgA binding with an ORF.

- "consensual motif" should be "consensus motif".
This has now been corrected

- Line 396, "insides" should be "insights".
This has now been corrected

Reviewer #2 (Comments for the Author):

The authors have answered all of my concerns. Just one minor mistake:

line 357 please correct reference. It says #8, should be #7
This has now been corrected

April 24, 2020

Dr. Loic Coutte
INSERM
microbiology
U1019-CIIL-Institut Pasteur de Lille
1 rue du Pr Calmette
Lille 59021
France

Re: mSystems00208-20R1 (Combined RNAseq and ChIPseq analyses of the BvgA virulence regulator of Bordetella pertussis)

Dear Dr. Loic Coutte:

Your manuscript has been accepted, and I am forwarding it to the ASM Journals Department for publication. For your reference, ASM Journals' address is given below. Before it can be scheduled for publication, your manuscript will be checked by the mSystems senior production editor, Ellie Ghatineh, to make sure that all elements meet the technical requirements for publication. She will contact you if anything needs to be revised before copyediting and production can begin. Otherwise, you will be notified when your proofs are ready to be viewed.

As an open-access publication, mSystems receives no financial support from paid subscriptions and depends on authors' prompt payment of publication fees as soon as their articles are accepted. You will be contacted separately about payment when the proofs are issued; please follow the instructions in that e-mail. Arrangements for payment must be made before your article is published. For a complete list of **Publication Fees**, including supplemental material costs, please visit our website.

Corresponding authors may join or renew ASM membership to obtain discounts on publication fees. Need to upgrade your membership level? Please contact Customer Service at Service@asmusa.org.

Thank you for submitting your paper to mSystems.

Sincerely,

Mark Mandel
Editor, mSystems

Journals Department
American Society for Microbiology
1752 N St., NW
Washington, DC 20036
E-mail: peerreview@asmusa.org

Phone: 1-202-942-9338

Supplemental Material: Accept
Supplemental Material: Accept
Supplemental Material: Accept
Supplemental Material: Accept
Supplemental Material: Accept
Supplemental Material: Accept
Supplemental Material: Accept
Supplemental Material: Accept
Supplemental Material: Accept
